# How young adults in the United States understand and conceptualise ultra-processed foods

**DOI:** 10.1017/jns.2026.10090

**Published:** 2026-03-30

**Authors:** Alexandra B. Larcom, Ingrid E. Lofgren, Matthew J. Delmonico, Amanda Missimer, Kathleen J. Melanson

**Affiliations:** 1 Department of Nutrition, University of Rhode Island, USA; 2 Department of Kinesiology, University of Rhode Island, USA

**Keywords:** Consumer perceptions, food processing, mixed-methods, UPF, nutritional quality

## Abstract

Consumer understanding of ultra-processed foods (UPFs) is poor, and no consensus definition exists. This study examines how young adults in the United States (US) define UPF and their ability to differentiate UPF from non-UPF of varying nutritional quality (NQ). In a mixed-methods survey of young adults (18–39 years) living in the US for ≥1 year, respondents defined UPF, identified whether 24 foods were UPF or not using images with front and back of package information, and answered demographic questions. Foods were categorised using NOVA for processing and Food Compass for NQ. They included a high NQ non-UPF, low NQ non-UPF, high NQ UPF, and low NQ UPF item from six food groups: fruits, vegetables, dairy, grains, protein, and snacks/sweets. Concepts used to define UPF were reported as number of respondents mentioning each in their definition. A score of correct answers out of 24 was calculated. The sample of 422 adults, mean age 26.0±6.7 years, was predominantly white (82%), female (74%), and from the Northeast (82%). Thirty concepts were identified to define UPF. The top concepts were food containing additives, preservatives, colours/dyes, or natural or artificial flavours (*N* = 105), containing non-natural/artificial ingredients or food (*N* = 98), being highly processed/processed in multiple steps (*N* = 95), being altered, manipulated, or modified (*N* = 87), and having low nutritional value/nutrients removed (*N* = 75). The mean score was 16.0±3.6 (67%) foods. These results suggest limited consensus on how young adults define UPF. Studies in more diverse populations are needed, but consumers may benefit from a clear definition of UPF.

## Graphical abstract

**Figure:**
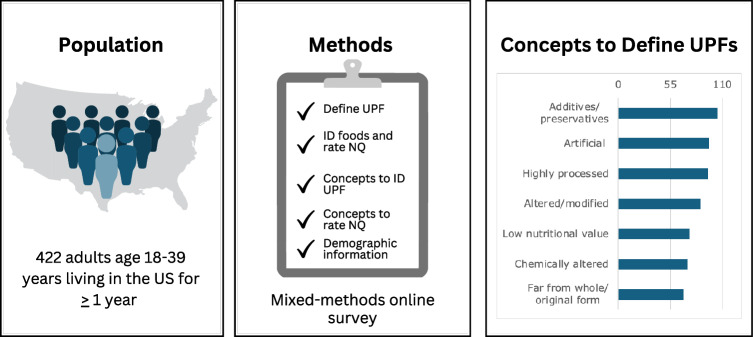

## Introduction

Multiple definitions have been used in the scientific literature to define ultra-processed foods (UPFs).^(1–7)^ However, most are complex and designed for research rather than communicating with consumers, and the scientific community still lacks consensus on how to define UPFs. Four themes for defining highly or UPF were identified from the seven different classification systems described in the scientific literature: ingredient formulation,^(3,4,6,7)^ alterations to the food matrix,^(2,4–6)^ setting of processing (homemade vs industrial formulations),^(4–7)^ and nutritional content.^(1–3,7)^ Some definitions use one factor such as alterations to the food matrix or nutrient content (e.g. saturated fat or sodium), while others use a combination of factors. The most widely used in the scientific literature is the NOVA classification system (not an acronym) developed by Monteiro and colleagues^(6,8)^ in Brazil in 2009, which defines UPF based on ingredient formulation and alterations to the food matrix, as well as an industrial setting.^(6,9)^

According to NOVA,^(6)^ UPFs are defined as industrialised products containing little or no whole foods, including frozen and prepared meals, packaged snacks and desserts, carbonated soft drinks, and processed meats. NOVA employs four groups to categorise foods by the extent and purpose of processing. These groups are 1) Unprocessed or minimally processed foods, including edible parts of plants and animals, as well as natural foods that are processed by methods such as removing unwanted parts, grinding, boiling, pasteurising, refrigeration/freezing, or fermenting, in the absence of any additional ingredients; 2) Processed culinary ingredients obtained from group 1 foods or nature, including salt, sugar, honey, butter, and vegetable oils to cook, season, or prepare group 1 foods; 3) Processed foods, which consist of foods or preparations combining category 1 and 2 foods including smoking, salting, curing, or canning the purpose of which is to increase the palatability and durability of group 1 foods; and 4) UPFs, defined as industrial formulations including ingredients not commonly used in culinary preparations or additives to imitate group 1 food qualities or mask undesirable qualities of the resulting food product.

In epidemiological studies, consuming a high percentage of energy from UPF has been associated with poor diet quality using multiple indices including the Healthy Dietary Adherence Score,^(10)^ alternative Mediterranean diet index, and Dietary Approaches to Stop Hypertension score,^(11)^ as well as with less healthy dietary patterns identified through principal component analysis.^(12)^ UPFs tend to be less nutrient-dense^(13,14)^ and contain more nutrients associated with non-communicable diseases^(15)^ compared to minimally processed or processed foods. However, there is wide variation in nutritional quality (NQ) within all four NOVA groups.^(16)^ Concerns about the connection between UPF and diet quality have prompted several countries, such as Brazil, Belgium, Israel, and the United States to incorporate recommendations about food processing levels into their national dietary guidelines.^(17,18)^ However, most of the classification systems currently in use, including NOVA, were not designed for consumers. Currently, there is no consumer-facing definition of UPFs in the United States (US), though the United States Department of Agriculture and Food and Drug Administration are working on a joint effort to develop a definition.^(19)^

The extent to which consumers understand recommendations surrounding UPF intake is poorly understood and has been limited primarily to studies in Latin America and Europe. Qualitative data from several of these studies^(20–23)^ shows consumers lack consensus on and use a wide range of concepts to define UPF. Further, evidence suggests consumers^(24–27)^ and nutrition professionals^(28)^ are inconsistent in their ability to identify UPFs, even though they largely report being familiar with the concept.^(24–27)^ In one study, the correlation between self-reported knowledge and tested knowledge suggests even among those who report knowledge about UPF, confusion remains.^(24)^ Evidence also suggests consumers^(22,25,29)^ and health professionals^(30)^ consider UPFs to be less healthy than unprocessed foods. In most countries, UPF consumption is highest among young adults (ages 18–39 years)^(31–33)^ compared to other adult age groups. In the US, young adults tend to have poorer diet quality than mid-life and older adults,^(34–36)^ and diet quality is a primary modifiable risk factor for health outcomes in this age group.

Except for one study in which French nutrition professionals reviewed one to two lists of 120 foods^(28)^ and one in which Brazilian consumers reviewed a list of 31 foods,^(37)^ most studies explored how consumers understand and identify UPFs by providing participants with a small selection of just 10–13 foods to categorise.^(24,25)^ This limits the range of food types, processing levels, and NQ of foods represented. Further, no study focusing on UPFs has fully explored the information consumers use to decide if a food is UPF or not and to rate its NQ. Amid government efforts to develop a uniform definition for UPF^(19)^ it is important to understand how the population perceives and understands the concept of food processing and UPF. The objectives of this study are (1) to describe the concepts young adults in the US use to define UPF, (2) to assess how well young adults in the US can distinguish UPFs from non-UPFs, and (3) to identify key concepts that young adults in the US use to make decisions about individual foods’ processing level and NQ.

## Materials & methods

### Participants and recruitment

Data were collected using an anonymous mixed-method, web-based survey (Qualtrics, Provo, UT). This study was conducted according to the guidelines laid down in the Declaration of Helsinki and all procedures involving research study participants were approved by the University of Rhode Island’s Institutional Review Board (IRB #2323-069). Written informed consent was obtained from all participants. Young adults living in the US for at least one year were recruited for the study. This study defined young adults as those ages 18–39 in alignment with the American Heart Association.^(38)^ To capture approximately 14% of adults living in the US who are foreign-born and would be part of the target audiences for dietary guidelines about UPFs, we included living in the US for at least one year to the inclusion criteria.^(39)^

Participants were recruited using convenience and snowball sampling from multiple sources, including participants in an ongoing study, university students, faculty, and staff, the surrounding community, and via social networks and social media outreach. 2020 US census data^(40)^ for adults ages 20–39 (data were not available for 18–39) and Qualtrics sample size calculator^(41)^ were used to calculate a proportionate sample of young adults relative to the general population. Based on a total population of 87,879,000 young adults and using a 95% confidence level and a 5% margin of error, the target sample size was 385 respondents.

### Data collection

The survey was designed for this study and used questions and methodologies from previously published research on UPF conceptualisation in other countries.^(20,21,24,25,28)^ The survey included three sections. In Section 1, participants were asked to define UPF and were provided a comment box to respond freely with no word or character limit, using similar question phrasing as Aguirre et al.^(20)^ and Ares et al.^(21)^ In Section 2, participants were provided images of 24 food items commonly consumed in the US and were provided images of the front of the package for all foods and the nutrition facts label and ingredients list for all foods except fresh produce (cherry tomatoes and grapes) for which that information is not provided on the package. Participants were asked to classify the food as ‘UPF’ or ‘non-UPF’ similar to methodologies used in Brazil,^(24,25)^ Italy,^(25)^ the Netherlands,^(25)^ Spain,^(26)^ and the UK,^(27)^ and they were also asked to rate the healthfullness of the food on a scale from 1 (lowest NQ) to 10 (highest NQ). In Section 3, participants were asked to rate their confidence level in categorising foods as UPF or non-UPF and to describe in free response with no word/character limit the factors they used to decide whether a food was UPF or non-UPF and to rate its healthiness. The full survey can be found in **supplemental materials**. Data were collected between October 2023 and April 2024.

### Food selection

Six food groups (fruits, vegetables, grains, dairy, proteins, and snacks/sweets) were selected based on the groups used in ‘What We Eat in America’.^(42)^ A high NQ non-UPF, low NQ non-UPF, high NQ UPF, and low NQ UPF food was selected for each of the six food groups for a total of 24 food items. While other studies used multiple processing levels, a binary classification was selected for the present study because NOVA was not designed for consumer education, and evidence suggests UPF is the NOVA category most associated with poor diet quality.^(11)^ Foods were classified as UPF or non-UPF according to the NOVA classification. Non-UPF foods included minimally processed and processed foods from NOVA groups 1 and 3, while UPFs included foods from NOVA group 4. The NQ of selected food items was determined using the Tufts University Food Compass score.^(43)^ Food Compass scores are calculated using an algorithm based on 54 individual attributes across nine domains (nutrient ratios, vitamins, minerals, food-based ingredients, processing, additives, specific lipids, fibre and protein, and phytochemicals). Scores range from 1–100, with 1 being the least and 100 being the most healthful. In this study, Food Compass scores ranged from 1 (unsalted pretzels) to 100 (cherry tomatoes), with high NQ foods ranging from 60 (veggie burger) to 100 (cherry tomatoes) and low NQ foods from 1 (unsalted pretzels) to 51 (applesauce). A full list of foods, food images, and Food Compass scores are available in **supplemental materials**.

### Qualitative analysis

Responses to all three qualitative questions were uploaded to ATLAS.ti software (ATLAS.ti Scientific Software Development GmbH, Berlin, Germany) and coded by a graduate student who holds a registered dietitian-nutritionist credential using inductive coding. There was no limit to the number of concepts that could be assigned to each response, and concepts were assigned a code just once per response even if they were mentioned more than once in that response. For example, if a respondent mentioned both preservatives and dyes, the code ‘contains additives, preservatives, colours, flavours, GMOs, or hormones’ was assigned just once. Two additional professors (PhD-level registered dietitians) reviewed the codes, and all three worked together to combine codes until there was no duplication of concepts.

### Demographic variables

Following Section 3 of the survey, participants were asked to provide their age and to select their gender, race/ethnicity, education level, and current field of occupation from a list of provided options. Fields of occupation were identified and grouped by industry, and to identify respondents who may (e.g. nutrition and health professions) or may not (e.g. engineering and computer science) have higher knowledge about UPFs and NQ based on their profession.

### Statistical method

Descriptive statistics were reported using means with 95% confidence limits for continuous variables and frequencies and percentages for categorical variables. The age range of respondents spanned 22 years and covered a wide range of life stages therefore the sample was further stratified by age into two groups to capture emerging adults (aged 18–29 years)^(44)^ and older young adults (aged 30–39 years). Missing data were excluded from analysis.

The primary outcome was to identify key terms and concepts used by young adults in the US to define UPFs. Text analysis was conducted using ATLAS.ti software to identify keywords, phrases, and concepts used to define UPF. Code co-occurrence analysis was conducted to quantify the number and percentage of respondents who mentioned each concept. The secondary aim was to assess participants’ ability to distinguish UPF from non-UPF, reported as a score of correct answers out of the 24 food items. The variable score was assessed for normality using the Kolmogorov-Smirnov test. ANOVA with Tukey’s post hoc follow-up was run to examine associations between scores and demographic variables age group, gender, race/ethnicity, education, field of work/study, and region. Exploratory outcomes were (1) identifying which foods participants rated as high or low NQ, (2) identifying factors that inform consumers’ decisions about whether foods are UPF or not, and (3) identifying factors that inform how consumers rate food based on NQ. Text analysis was used to identify keywords, phrases, and concepts, which were reported as frequencies and percent of participants mentioning them for both factors that inform decisions about UPF classification and factors that inform NQ ratings.

## Results

**Figure 1.**
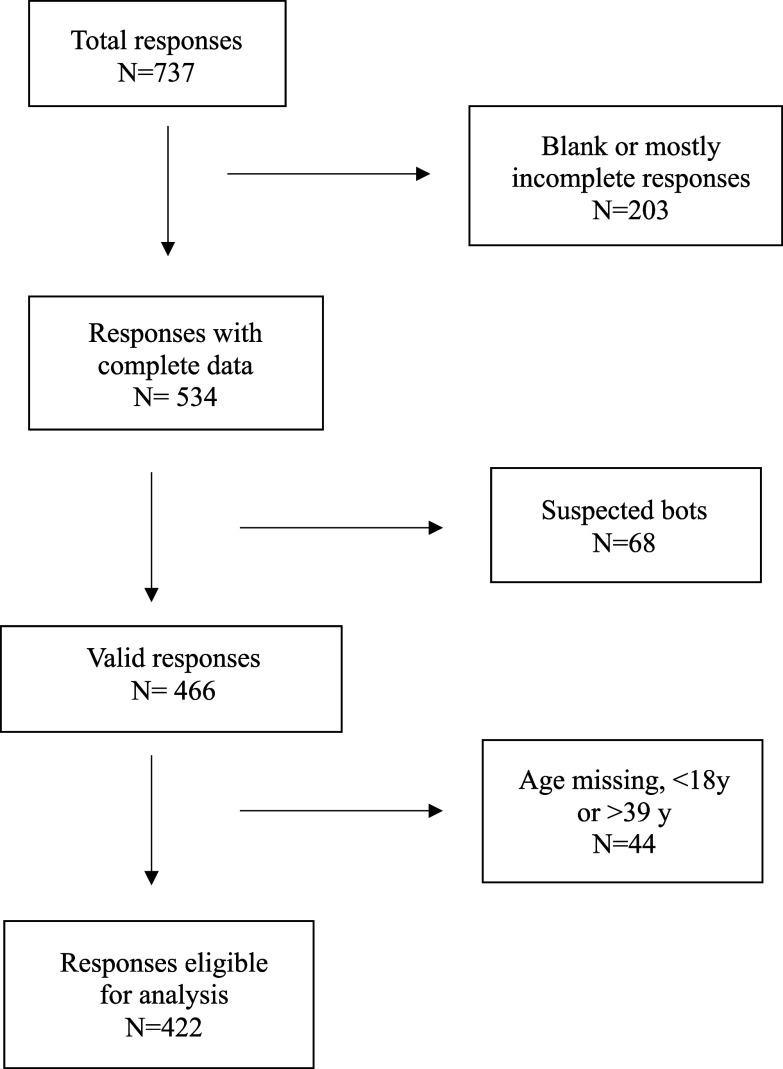
The participant flowchart.

A total of 737 respondents entered the survey using a Quick Response code or anonymous survey link. Figure 1 shows the participant flowchart. After eliminating blank (*n* = 131) and mostly incomplete (*n* = 72) responses, suspected bots (*n* = 68), and respondents who did not report their age (*n* = 14) or were outside the age range of 18–39 years (*n* = 30), the final sample was *N* = 422. Suspected bots were defined as responses with multiple identical qualitative answers within a short time (for this survey, <5 minutes).^(45)^

### Demographic variables

The sample had a mean age of 26.0 ± 6.7 years, with the majority falling in the younger (18–29 years) age range (*n* = 286, 67.8%). The sample was primarily female (74%), Caucasian (82%), and from the northeast or mid-Atlantic (82%). Table 1 describes participant characteristics.

**Table 1.** Participant characteristics

Variable	*N* = 422
Age, mean (SD)	26.0 (6.7)
Gender	
Female	313 (74%)
Male	104 (25%)
Non-binary	5 (1%)
Race *n* (%)	
White/Caucasian	345 (82%)
Black/African American	10 (2%)
Hispanic/Latino	17 (4%)
Asian/Pacific Islander	27 (6%)
More than one race	16 (4%)
Other/did not answer	7 (2%)
Region *n* (%)	
Northeast/Mid-Atlantic	348 (82%)
South/Southeast	50 (12%)
West/Southwest	12 (3%)
Midwest	12 (3%)
Field of work/study* *n* (%)	
Healthcare practitioners	53 (13%)
Life/physical sciences	61 (15%)
Nutrition/food sciences	47 (11%)
Accounting/business	21 (5%)
Sales/marketing	23 (6%)
Computer science/math	22 (6%)
Education	23 (6%)
Social sciences	36 (9%)
Other	129 (31%)
Did not answer	17 (4%)
Education *n* (%)	
High school	18 (4%)
Enrolled in college	168 (40%)
Some college	11 (3%)
Completed college	107 (25%)
Master’s degree	90 (21%)
Terminal degree	28 (7%)

* *N* = 415.

### Concepts used to define UPFs

A total of *n* = 420 respondents defined UPFs. A total of 30 concepts were identified and categorised into six themes. The top concepts mentioned in order were: food containing additives, preservatives, colours/dyes, or natural or artificial flavours, containing non-natural or artificial ingredients or food, food being highly processed or processed in multiple steps, food being altered, manipulated, or modified, and food having low nutritional value or nutrients removed. Table 2 lists each concept identified by theme as a percent of respondents mentioning the concept.

**Table 2.** Concepts used to define UPFs by theme

Theme	Concept	Percent^a^
Ingredients	Contains additives, preservatives, colours, flavours, GMOs, or hormones	25.0
	Familiarity with food or ingredients, or ability to pronounce ingredients	6.2
	Has a lot of ingredients or added ingredients	6.0
	Ingredients not available/accessible in a home kitchen	3.3
	Contains specific types of ingredients (e.g. syrups, oils)	2.4
Natural or whole	Non-natural, artificial, or fake food or ingredients	23.3
	Food/ingredients not or are significantly changed from whole, real, or original form	16.4
Processing	Highly processed or processed in multiple steps	22.6
	Altered, manipulated, or modified	20.7
	Contains chemicals or is chemically altered	17.4
	Industrial, factory, or commercially processed/produced	14.5
	Based on the NOVA classification system	0.2
Nutritional value	Low nutritional value/unhealthy or stripped of nutrients	17.9
	Presence of specific nutrients, e.g. sugar and sodium, and nutrient density	6.9
Food qualities	Having a long shelf life	11.2
	Processed to improve taste, texture, or appearance	6.4
	Packaged or branded	6.2
	Low cost or cheap to produce or buy	2.6
	Food or ingredients are not organic	1.0
	Designed to encourage consumption	0.7
Food type	Fast food, junk food, or fried foods	3.3
	Pre-prepared, ready to eat, or frozen foods	2.1
	Food type, e.g. chips, sodas, syrups	1.2
Other	Don’t know	0.7
	Gross or poison	0.7
	Food not available locally	0.5
	Unnecessary processing or ingredients	0.5
	Located in the centre of the grocery store	0.2
	Not available for most of human history	0.2

a Because respondents could mention more than one concept, the percentage of respondents mentioning each concept exceeds 100%.

### Ability to identify UPFs

Respondents correctly identified a mean of 16.0 (CI 15.7–16.3) out of 24 foods as UPF or not UPF using the NOVA definition. The variable score was not normally distributed (*p* < 0.01), but skewness (−1.1) and kurtosis (2.8) were within acceptable ranges, so parametric tests were performed.^(46)^ The majority of respondents were at least moderately confident in their ability to distinguish UPFs from non-UPFs, with 59% reporting moderate confidence, 29% reporting low confidence, and 12% reporting high confidence. Respondents reporting high confidence scored better (*M* = 18.0, CI 17.2–18.0) than those with moderate (*M* = 15.9, CI 15.5–16.4) or low (*M* = 15.3, CI 14.5–16.1) confidence (*p* < 0.01).

In subgroup analysis by demographic variables, significant differences in score were found for gender, region, age group, and education. No differences were observed for race and field of work/study. Females scored higher than males (*M* = 16.5 ± 3.2 vs *M* = 14.5 ± 4.2, *p* < 0.01), individuals from the Midwest scored higher than those from the Northeast (*M* = 18.7± 2.5 vs *M* = 15.8 ± 3.6, *p* = 0.02), respondents age 30 and over scored higher than those ages 18–29 (M = 16.8 ± 2.9 vs *M* = 15.6 ± 3.8, *p* < 0.01), and people who had completed college or a masters degree scored higher than those who were enrolled in or had attended but left college (*M* = 16.6 ± 3.1 and 16.9 ± 3.0 vs 15.4 ± 3.8 and 13.1 ± 6.1, *p* < 0.01**)**.

Respondents identified 22 concepts that informed their decision about whether a food was UPF or not (Table 3). Most respondents relied on ingredient type, quality, or simplicity (57.0%). Other concepts mentioned by at least 10% of respondents include the use of additives, preservatives, colours, or artificial or natural flavours (20.0%), the number of ingredients or length of the ingredients list (19.1%), specific nutrients (15.2%), whether food or ingredients were natural or artificial/fake (10.9%), and based on packaging, branding, or marketing (10.4%).

**Table 3.** Concepts used to identify UPF foods by theme

Theme	Concept	Percent^a^
Ingredients	Ingredient type (e.g. oils, syrups), quality, or simplicity	59.9
	Presence of additives, preservatives, colours/dyes, flavours, chemicals, GMOs, or hormones	24.2
	Number of ingredients/length of ingredients list	19.1
	Containing added or extra ingredients	4.1
Nutritional content	Presence of specific nutrients, e.g. sodium, sugar, fat	15.2
	Using information on the front of package or nutritional facts label	5.1
	Nutritional value, energy/nutrient density, or macro and micronutrient balance	2.7
Natural or whole	How far from or whether food or ingredients were in whole, original, or natural form	15.2
	Whether food or ingredients were naturally occurring or artificial/fake	10.9
Food qualities	Appearance – packaging, branding, marketing	11.1
	Extent or length of shelf life or freshness	5.8
	Type of food, e.g. fruit/vegetable or snacks/sweets	2.7
	Whether food was organic or not	1.9
	How food was prepared/sold (e.g. frozen, convenience store)	1.0
Personal factors	Familiarity with the ingredients/food or ability to pronounce ingredients	8.2
	Prior knowledge or personal assumptions, bias, or experience	6.5
	How food tastes	0.5
	Don’t know, guessed, or used Google	0.5
Processing	Processing level – food/ingredients highly processed or processed in multiple steps	4.6
	Commercially/mass produced, made in a factory	2.4
	Ability to access ingredients or prepare food at home, or to access foods before modern times	2.7
	Based on the NOVA classification system	0.2

a Because respondents could mention more than one concept, the percentage of respondents mentioning each concept exceeds 100%.

### Nutritional quality ratings and concepts

**Figure 2.**
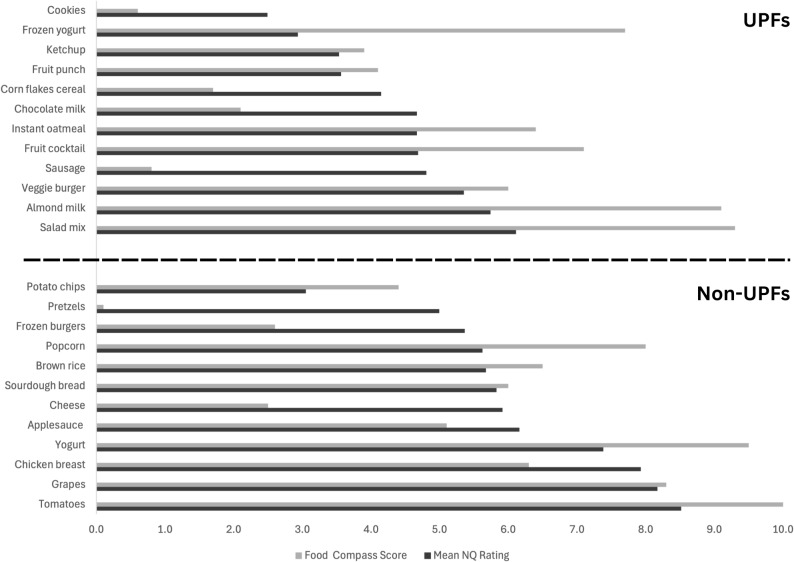
NQ ratings by processing category compared to food compass scores.

The five foods respondents rated as highest in NQ were all non-UPF – tomatoes, grapes, chicken breast, yogurt, and applesauce – while the bottom five foods were all UPF – cookies, frozen yogurt, ketchup, and fruit punch – except for potato chips. Figure 2 depicts NQ ratings in order from lowest to highest, colour-coded by processing level and contrasted with the Food Compass scores used to determine the NQ of selected foods. Agreement was observed between NQ ratings and food processing, where foods rated lowest for NQ were UPF and the foods rated highest NQ foods were non-UPF except for potato chips (low NQ non-UPF) and almond milk and salad mix (high NQ UPF). There was more agreement between NQ ratings and Food Compass scores for non-UPF foods than for UPF foods. Among non-UPF foods, respondent NQ ratings and Food Compass scores were similar except for popcorn and plain yogurt, which respondents rated lower than Food Compass scores, and pretzels and applesauce, which respondents rated higher than Food Compass scores. The opposite was true among UPF foods, where only ketchup, fruit punch, and veggie burgers showed respondent ratings similar to their Food Compass scores. Most other UPF foods were rated lower NQ by respondents compared to their Food Compass scores except for cookies, Corn Flakes cereal, chocolate milk, and sausages, which respondents rated higher NQ compared to Food Compass.

Respondents identified 26 concepts used to rate a food’s NQ (Table 4). The most frequently mentioned was the presence of specific nutrients such as sodium, saturated fat, dietary fibre, and sugar (46.1%). Other top considerations include ingredient type (e.g. oils or syrups), quality, or simplicity, prior knowledge or personal assumptions or biases about foods, nutritional quality and nutrient or energy density, and the information provided on the front of package or nutrition facts label.

**Table 4.** Concepts used to rate foods’ NQ by theme

Theme	Concept	Percent^a^
Nutritional value	Presence of specific nutrients, e.g. sodium, sugar, fibre	46.1
	Nutritional quality, energy/nutrient density	12.4
	Nutrient balance or contribution to diet quality	6.3
	Health effects, satiety, or how the body feels after eating	1.7
	Alignment with specific diet/dietary pattern	0.7
Ingredients	Ingredients, type (e.g. oils or syrups), quality, or simplicity of ingredients	28.5
	Number of ingredients/length of ingredients list	7.6
	Presence of additives, preservatives, colours/dyes, natural or artificial flavours, or chemicals	6.3
	Added or extra ingredients	1.0
Personal or social factors	Prior knowledge or personal assumptions, bias, or experience	14.9
	Familiarity with ingredients/food or ability to pronounce ingredients	2.2
	Stereotypes from media or society	1.0
	How food tastes	0.2
Packaging/appearance	Front of package or nutrition facts label	11.2
	Packaging, branding or marketing	2.4
Food qualities	Type of food, e.g. fruit/vegetable or snacks/sweets	8.0
	Extent of shelf life or freshness	2.4
	Whether food was organic or not	1.5
	Clean, pureness, and wholesomeness	1.2
Whole or natural	Whether food or ingredients are naturally occurring or artificial/fake	6.6
	How far from or whether food or ingredients are whole or in original or natural form	5.9
Processing	Processing level or number of steps required	6.3
	Commercially/mass produced or made in a factory	0.5
Other	Convenient or found in a convenience store	0.5
	Don’t know/guessed or used Google	0.5
	No definition – all foods can be healthy	0.5

a Because respondents could mention more than one concept, the percentage of respondents mentioning each concept exceeds 100%.

Some overlap was observed between concepts used to distinguish between UPF and non-UPF foods and to rate foods’ NQ. Both the presence of specific nutrients (e.g. sodium or sugar) and ingredient type, quality, or simplicity were mentioned by over 15% of respondents as both factors that helped them distinguish between UPF and non-UPF foods and to rate foods’ NQ. The presence of specific nutrients was mentioned by 46.1% of people to rate foods’ NQ and 15.2% to distinguish UPFs and non-UPFs, and ingredient types, quality, and simplicity were mentioned by 59.9% of people to distinguish UPFs and non-UPFs and 28.5% of people to rate foods’ NQ. Other concepts mentioned by at least 5% of respondents for both UPF and NQ were the presence of additives, preservatives, colours or dyes, flavours, or chemicals (24.2% and 6.3%, respectively), the number of ingredients or the length of the ingredients list (19.1% and 7.6%), how far food or ingredients were from their original, whole, or raw form (15.2% and 5.9%), whether food or ingredients were artificial/fake or natural (10.9% and 6.6%), based on prior knowledge or personal assumptions or biases (6.5% and 14.9%, respectively), and using information on the front of package or nutrition facts label (5.1% and 11.2%).

## Discussion

This study is the first to document how young adults living in the US perceive and understand the concept of food processing and UPF. It also adds to the scientific literature because concepts related to NQ were included. Respondents identified 26 concepts to define UPFs, with over 20% of respondents mentioning specific ingredients like additives or preservatives, artificial or non-natural foods or ingredients, and food having been processed in multiple steps or altered/modified in some way. These findings differ from two similar studies in South American adults in which food having been highly processed or processed multiple times was the top concept for defining UPFs.^(20,21)^ Processing level or industrial processing was also the top choice for respondents in one study from the Netherlands, Italy, and Brazil, who selected industrial processing as the top answer in a multiple choice format, followed by artificial ingredients, genetically modified products, and the number of ingredients.^(25)^ Similarly, when given a binary choice, the majority of respondents to another survey disseminated in Brazil selected ‘foods with many processes performed by the food industry’ (78.2%) as opposed to ‘foods with many ingredients in their formulation’.^(24)^ However, in both this study and Uruguayan employees over age 18 surveyed by Ares et al.,^(21)^ having been highly processed or processed in multiple steps as well as including specific ingredients like additives or preservatives and having artificial or non-natural ingredients were among the top concepts mentioned in defining UPF. In contrast to Ares et al.^(21)^ and this study, Ecuadorian and Argentinian university students surveyed by Aguirre et al.^(20)^ demonstrated more consensus in their definition of UPFs, with 66.6% of respondents mentioning the top concept of highly processed or multiple processing steps (compared with 30.7% in Ares et al.^(21)^ and 25% in this study mentioning the top concept). Aguirre et al.^(20)^ may have found more consensus because that study included a more homogenous population (first-year University students) and had a smaller sample size (*N* = 181) compared to *N* = 2281 in Ares et al.^(21)^ and *N* = 420 in this study).

Four themes for defining highly or UPF were identified from the seven different classification systems described in the scientific literature. In this study, respondents considered all four of the factors used to define UPF in the scientific literature – ingredient formulation,^(4–7)^ alterations to the food matrix,^(2,4)^ setting of processing (homemade vs industrial formulations),^(4–7)^ and nutritional content.^(1–3,7)^ – in their definitions of UPF. Twenty-five per cent mentioned specific ingredients, 22.6% mentioned the extent of processing or the number of processing steps, 17.9% mentioned food having low nutritional value or removal of nutrients, and 14.5% mentioned industrial processing. This was similar to findings from Ares et al.,^(21)^ in which 30.7% mentioned processing and 26.4% mentioned specific ingredients, though in that study only 10% or fewer mentioned concepts related to nutritional value or quality. In contrast, most respondents in Aguirre et al.^(20)^ mentioned alterations to the food matrix (highly processed or industrial processing), and less than 10% mentioned concepts related to ingredients or nutritional value. The multiple definitions for ultra- or highly processed foods and results from these studies suggest that there is a lack of consensus as to how to define and distinguish foods by processing level for scientists and consumers.

Respondents in this study performed poorly in correctly distinguishing UPFs, with a mean score of 67% of foods correctly identified. This aligns with findings from a study in Brazil, where respondents scored 62% when asked to decide whether six foods were UPF or not and to answer four True/False questions.^(24)^ However, respondents in this study performed better than Brazilian consumers, who correctly identified just 32% of 31 foods as minimally versus ultra-processed using classifications from the Brazilian dietary guidelines. There was more consensus in this study among respondents as to how to distinguish UPF and non-UPF foods. Respondents mentioned 22 concepts to distinguish UPFs from non-UPFs, with the majority mentioning concepts related to ingredients. Over half (59.9%) mentioned ingredient types (e.g. syrups or oils), quality, or simplicity, and nearly a quarter (24.1%) mentioned specific ingredients like additives and preservatives. This is similar to a survey of Dutch consumers in which respondents reported paying attention to additives, the number of ingredients, and the type of ingredients (salt, sugar, fat) in processed foods.^(29)^ In a qualitative study including US and foreign-born elementary school parents, participants said they identified processed foods based on convenience or preparation method, packaging, added ingredients like chemicals or familiarity with ingredients, and healthfulness.^(22)^ These concepts were also mentioned in this present study but by fewer than 10% of respondents.

Respondents in this study also identified 26 concepts to rate foods’ healthiness. The presence of specific nutrients like sodium and sugar was mentioned most frequently, followed by ingredient types, numbers, or specific ingredients, and personal assumptions or biases. Other concepts included packaging and appearance, food qualities like the type of food or whether it was organic, whether the food was natural or whole, and processing level. Characteristics of foods – including specific nutrients, food type (e.g. fruits and vegetables), and processing – was also a top concept mentioned by Uruguayan adults participating in focus groups.^(47)^ When the discussion focused on UPFs, participants in that study considered health claims, packaging, ingredients, branding and logos, price, and country of origin to decide if the product was healthy. While they considered industrial processing and UPFs less healthy, they did not consider all UPFs to be unhealthy and acknowledged that some strategies to decide if a UPF was healthy or not might be unreliable.^(47)^ Similar themes have also emerged in studies that do not focus on UPF or food processing. In a review conducted by Plasek et al.,^(48)^ health claims or front-of-package labelling about the healthfulness of the food, packaging shape and colour, specific nutrients including sodium and fat, food type or product category (e.g. cereal vs cookies), whether a product was organic or natural, and taste or sensory appeal of the product were found to influence consumers perceptions of healthiness across multiple studies in multiple countries.

In the present study, overlap between concepts used to distinguish UPF and to rate foods’ NQ was observed, with specific nutrients like sodium or sugar and ingredient type, quality, or simplicity, mentioned by at least 15% of participants for both. The NOVA classification, the most widely used method for categorising foods by processing level in the scientific literature, does not consider NQ as part of its definition of UPFs, though several other definitions do define highly or ultra-processed foods based on NQ.^(1–3)^ While UPFs are more likely to have poorer nutrient density^(14)^ and NQ,^(13,49)^ there remains a wide variance in NQ across the NOVA categories.^(14)^ The foods chosen for this study reflected a wide range of NQ based on Food Compass scores in both UPF and non-UPF items. Despite this, there was agreement between NQ ratings and processing in this study, with respondents generally rating UPF items as lower NQ and non-UPF items as higher NQ. This aligns with findings from Bolhuis et al.,^(25)^ which observed a strong negative correlation between food processing and perceived healthiness among Dutch, Italian, and Brazilian consumers, and Bhawra et al.,^(30)^ in which Canadian nutrition professionals gave processed foods and UPFs lower NQ ratings than unprocessed foods.

### Strengths & limitations

This study has several key strengths. The mixed-methods study design provided in-depth information about how consumers define UPFs, as well as the specific factors they consider in distinguishing between products that are UPFs or not, and in assessing NQ. This study provided a relatively large sample size of qualitative responses, and thematic saturation was achieved. This study provided a wider range of food processing and NQ categories than previous studies,^(25,28)^ which more adequately represent the range of NQ available across food processing levels. This study also has some limitations. The sample of respondents was predominantly white, female, and located in the Northeast and Mid-Atlantic United States, and was more highly educated compared to the general US population. Results cannot be generalised to all young adults in the US, and more research is needed in more diverse populations, including more males, individuals without a college degree, and people living in a broader representation of US regions. It is possible that food selection could bias results, as there were 24 foods represented, and foods consumed by some cultural groups in the US were not included in the survey. Future studies should include regional and culturally diverse foods when evaluating UPFs to capture a broader representation of the US diet. The survey used in this study was not validated prior to use, however, questions and methodologies were adopted from previous research on consumer perceptions of UPFs.^(20,21,24,25)^ Data were collected from fall 2023 to spring 2024, and public discussion about UPFs, including from government agencies, has evolved since. It is possible consumers have increased understanding from ongoing discussions in media; however, there remains no government-sponsored definition of UPFs at the time of publication.

## Conclusions

Multiple definitions exist to define UPFs; however, most are complex and designed for research rather than communicating with consumers, and no consensus exists among scientists as to how to define UPFs. Findings from this and similar studies show that significant confusion persists among consumers as to how to define and identify UPFs, and what factors should be considered in deciding whether a food is UPF or not. Consumers in the US and globally would likely benefit from a clear, consumer-oriented definition and education about how to identify UPFs at points of purchase established by a trusted public health or government health organisation. More research is needed in diverse populations and in other age groups to inform the development of both a research-oriented and a consumer-facing definition for UPFs. A consensus definition can inform subsequent education materials to improve consumer knowledge about UPFs and help improve consumers’ diet quality.

## Supporting information

Larcom et al. supplementary material Larcom et al. supplementary material

## Supplementary material

The supplementary material for this article can be found at https://doi.org/10.1017/jns.2026.10090
