# Supplementary material for: How young adults in the United States understand and conceptualise ultra-processed foods
Source: J Nutr Sci. 2026 Mar 30;15:e21. doi: 10.1017/jns.2026.10090 (PMC13126058; doi:10.1017/jns.2026.10090)
Supplement: Larcom et al. supplementary material [file S2048679026100901sup001.pdf]

Supplementary Table 1 – Survey food items by food group, processing level, and NQ

Category	High NQ N-UPF	Low NQ N-UPF	High NQ UPF	Low NQ UPF
Fruit (WWEIA)	Grapes (83)<br><br>Welch's green seedless grapes	Applesauce (51)<br><br>Motts Applesauce	Fruit cocktail (71)<br><br>Delmonte 100% Juice Fruit Cocktail	Fruit juice beverage (41)<br><br>Mott's Mighty Flying Fruit Punch Juice
Vegetable (WWEIA)	Tomatoes (100)<br><br>NatureSweet Cherubs	Potato chips (44)<br><br>Utz Original Potato Chips	Asian salad (93)<br><br>Fresh Express Chopped Asian Salad kit	Ketchup (39)<br><br>Heinz tomato ketchup
Grains (USDA)	Brown rice (65)<br><br>Uncle Ben's Ready Rice	Sourdough bread (6)<br><br>When Pigs Fly Sourdough loaf	Instant oatmeal (64)<br><br>Great Value Maple & Brown Sugar Oatmeal	Cornflakes (17)<br><br>Kellogg's Corn Flakes
Protein (WWEIA)	Chicken breast (63)<br><br>Perdue All Natural Chicken Breasts	Ground beef (26)<br><br>Bubba Burgers	Veggie burger (60)<br><br>Morningstar Farms Garden Veggie	Pork sausage (8)<br><br>Johnsonville Mild Italian Sausage
Dairy	Plain yogurt (95)<br><br>Fage Greek Yogurt	Cheese (25)<br><br>Cabot Vermont Cheddar Cheese	Almond milk (91)<br><br>Almond Breeze Unsweet Almond Milk	Chocolate milk (21)<br><br>Horizon Chocolate Milk
Sweets/ snacks	Air Popcorn (80)<br><br>Boom Chika Pop Sea Salt popcorn	Unsalted pretzels (1)<br><br>365 Unsalted mini twists	Frozen yogurt (77)<br><br>Hood low fat frozen yogurt	Choc chip cookies (6)<br><br>Pepperidge Farm Montauk Milk Chocolate

Food item (Food Compass Score)

Supplementary Figures 1 – 24 – Survey food images

Fruits

Figure 1: Green seedless grapes

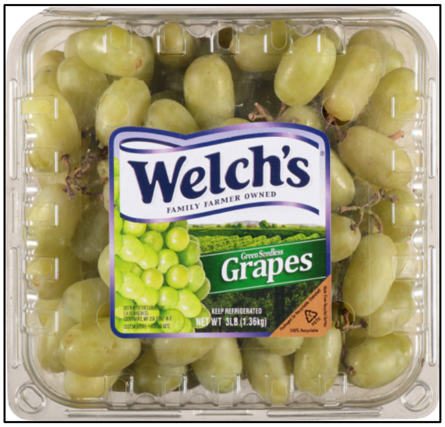

Figure 2: Applesauce

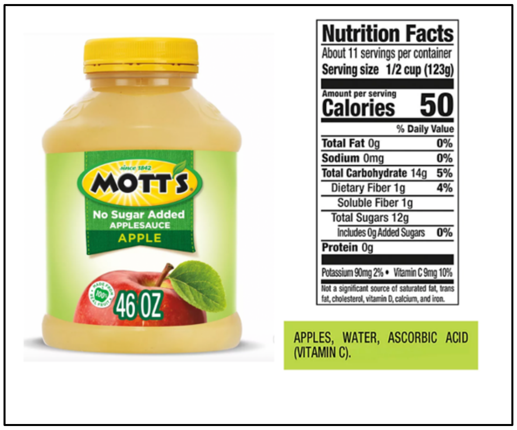

Figure 3: Fruit Cocktail

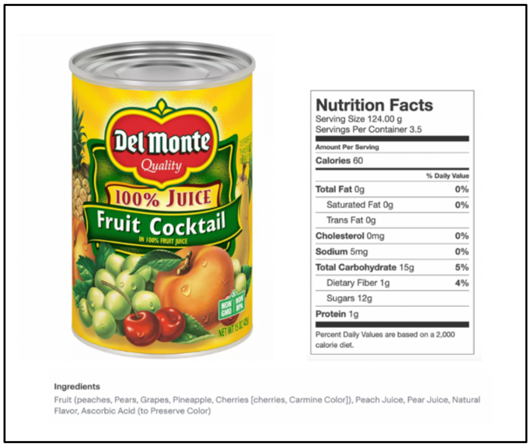

Figure 4: Fruit juice beverage

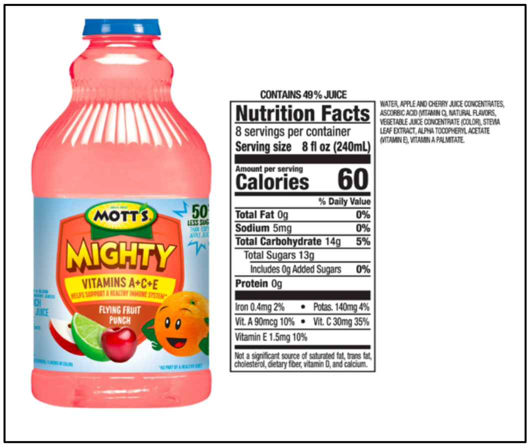

Figure 5: Cherry tomatoes

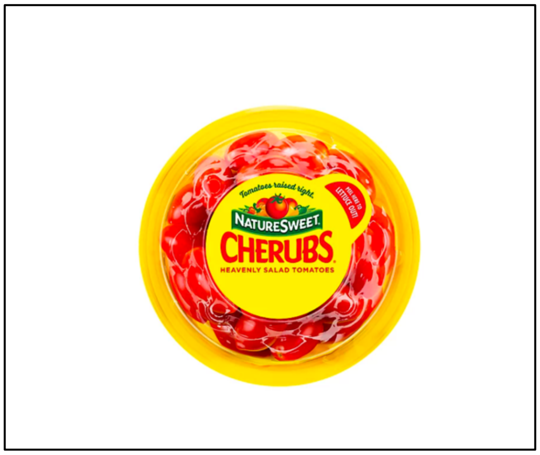

Figure 6: Plain potato chips

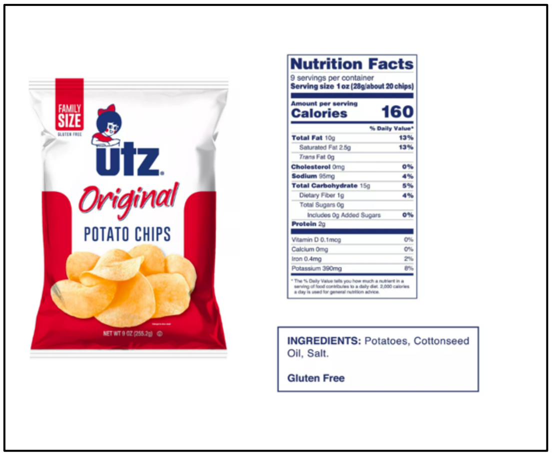

Figure 7: Asian salad mix

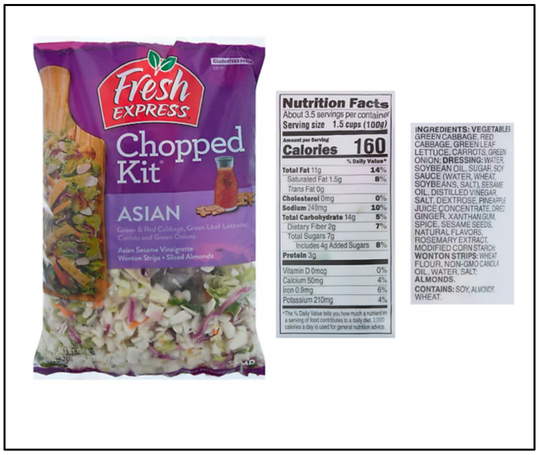

Figure 8: Tomato Ketchup

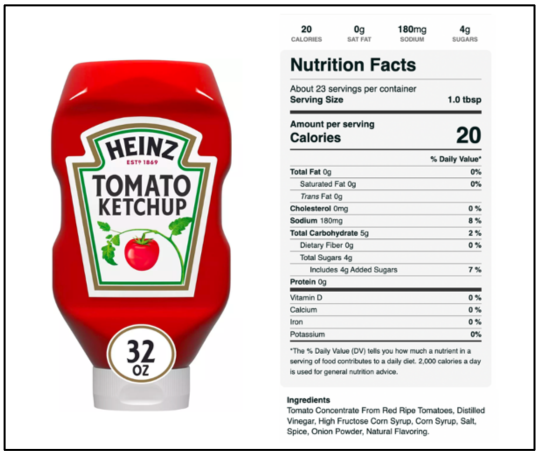

Figure 9: Brown rice

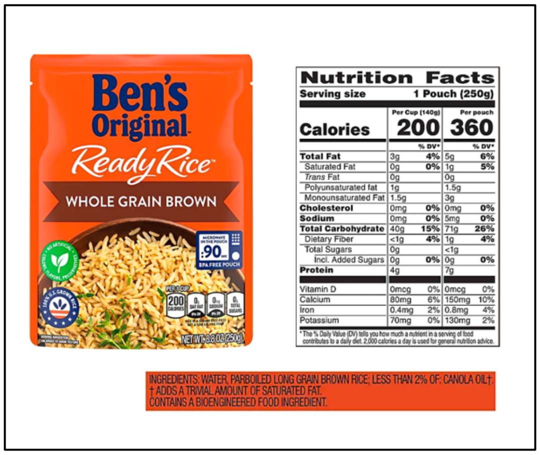

Figure 10: Sourdough bread

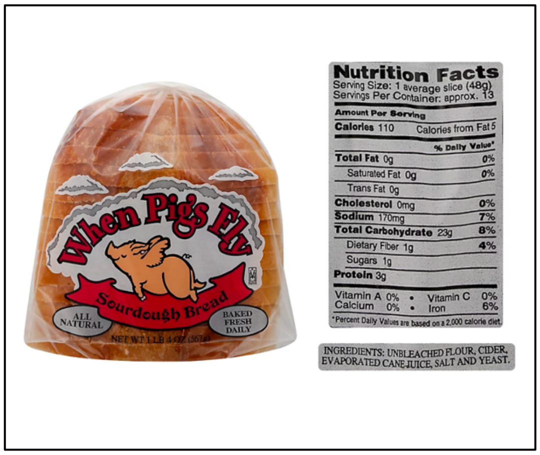

Figure 11: Maple brown sugar oatmeal

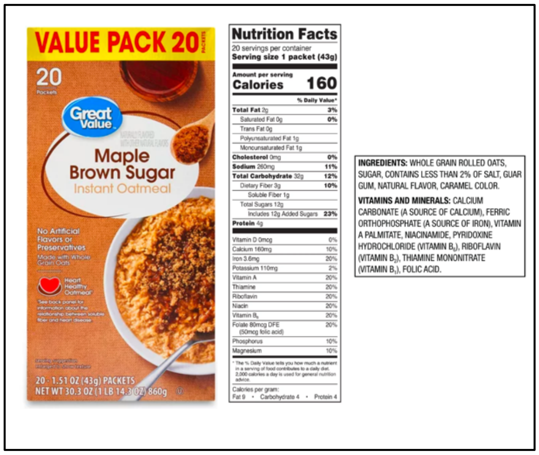

Figure 12: Corn Flaks cereal

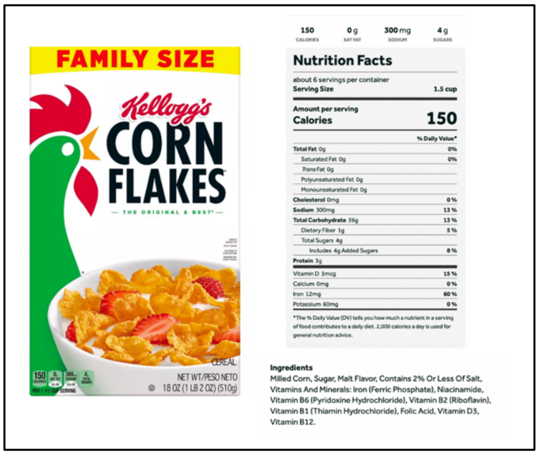

Figure 13: Chicken breast

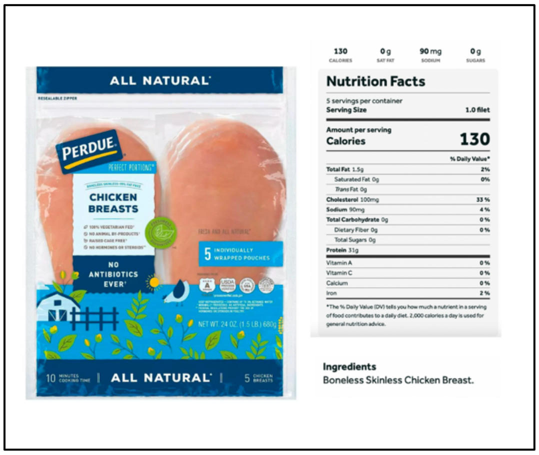

Figure 14: Frozen ground beef patties

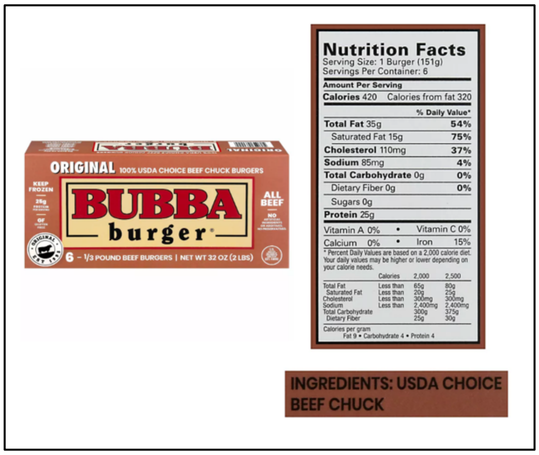

Figure 15: Garden veggie burger

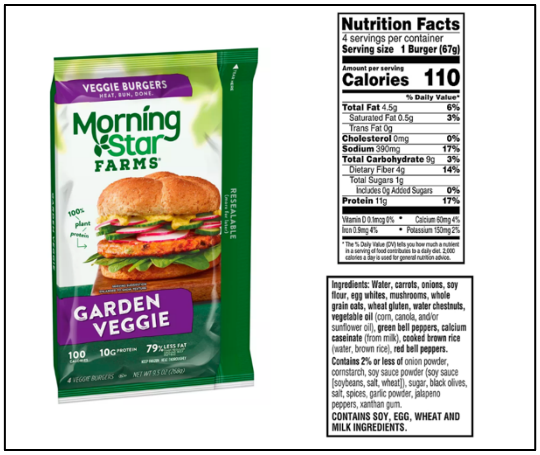

Figure 16: Pork sausage

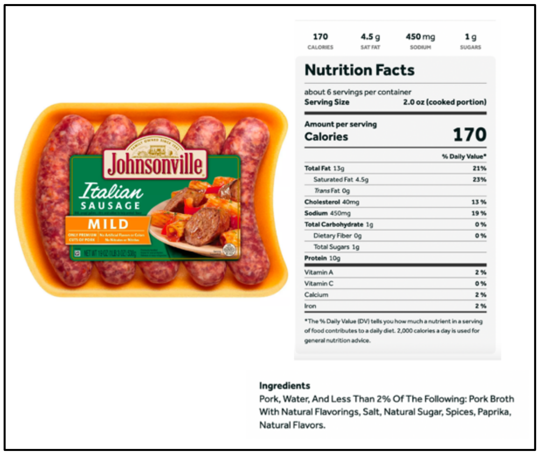

Figure 17: Plain Greek yogurt

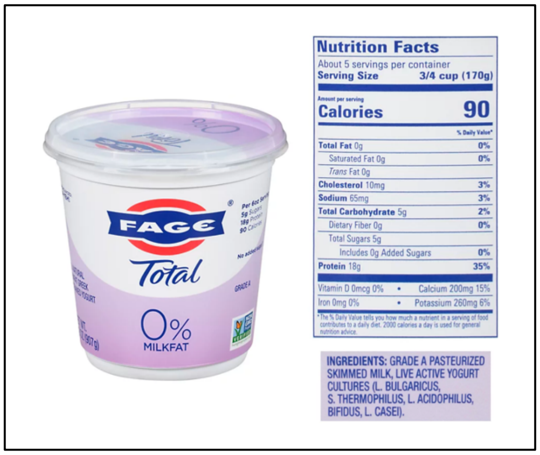

Figure 18: Extra sharp cheddar cheese

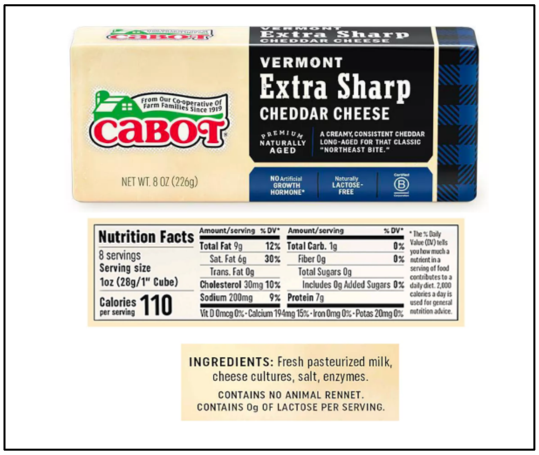

Figure 19: Almond milk

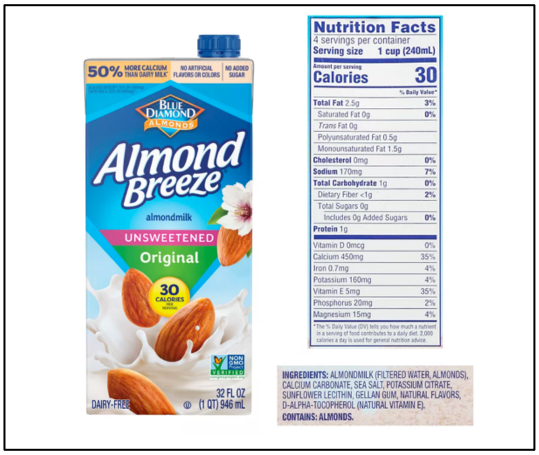

Figure 20: Chocolate milk

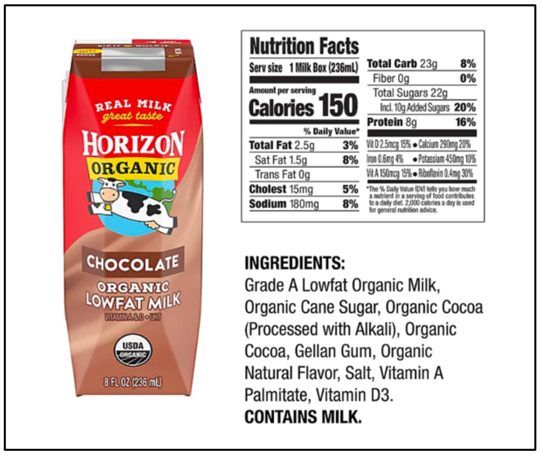

Figure 21: Plain salted popcorn

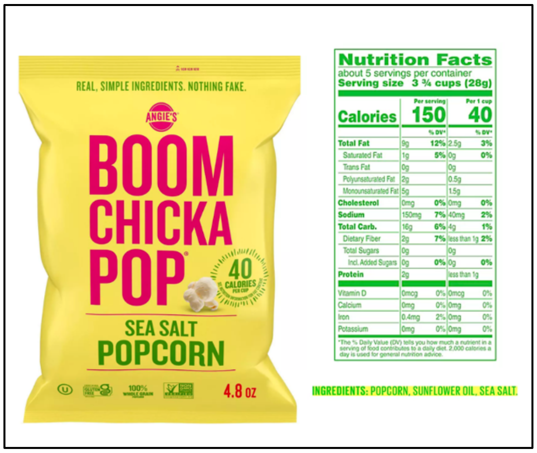

Figure 22: Unsalted pretzels

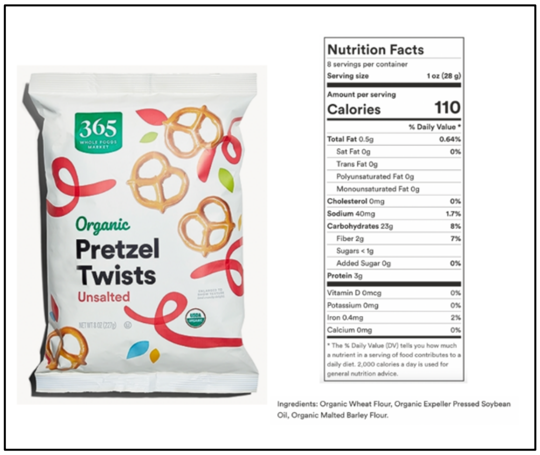

Figure 23: Frozen chocolate yogurt

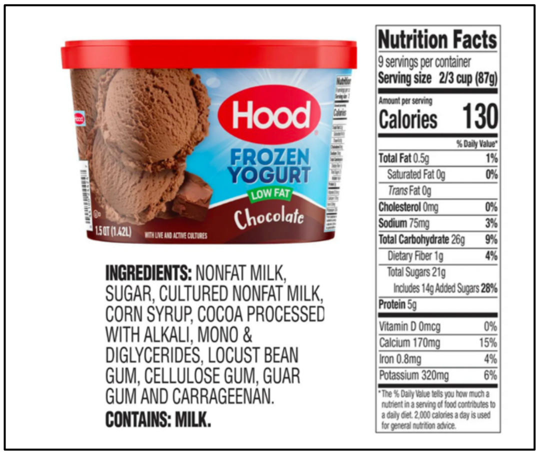

Figure 24: Chocolate chip cookies

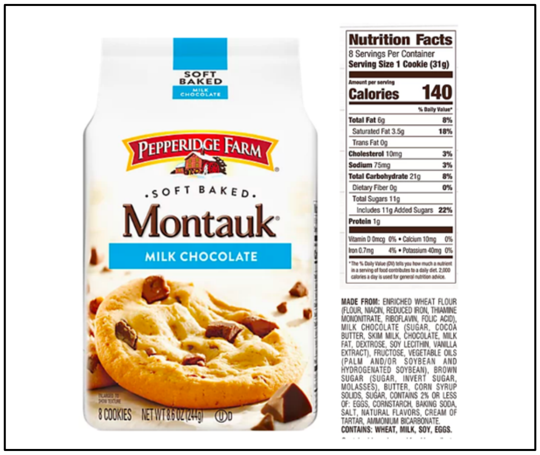

Qualtrics Survey

Consent

You are being asked to take part in a research study. The purpose of the research study is to examine how young adults living in the U.S. view, understand, and think about ultra-processed foods (UPFs). Please read the following before agreeing to be in the study. If you agree to be in this study, it will take you approximately 15 – 20 minutes to complete this survey. Questions will be asked about how you define UPF, and you will be asked to classify food items as UPF or not and to rank how healthy you think each food item is on a scale of 1 to 10. You will also be asked what factors or information you use to determine if a food is ultra-processed and to rate its healthiness. There are no known risks or benefits. All who complete the survey have the option to be entered in a drawing for one of four $50 Amazon gift cards. You will enter your email for this drawing in a separate form, and it will not be associated with your survey response.

Your responses will be strictly anonymous. The responses may be used as part of a doctoral dissertation, in scientific publications, and presented at academic conferences.

The decision to participate in this study is entirely up to you. You may refuse to take part in the study at any time without affecting your relationship with the investigators of this study or the University of Rhode Island (URI). Your decision will not

result in any loss of benefits to which you are otherwise entitled. You have the right not to answer any single question, as well as to withdraw completely from the survey at any point during the process.

You have the right to ask questions about this research study and to have those questions answered by me before, during or after the research. If you have questions about the study, at any time feel free to contact Kathleen Melanson from the Department of Nutrition at the University of Rhode Island at 401-874-4477.

Additionally, you may contact the URI Institutional Review Board (IRB) if you have questions regarding your rights as a research participant. Also contact the IRB if you have questions, complaints or concerns which you do not feel you can discuss with the investigator. The University of Rhode Island IRB may be reached by phone at (401) 874-4328 or by e-mail at researchintegrity@etal.uri.edu. You may also contact the URI Vice President for Research and Economic Development by phone at (401) 874-4576.

If you would like to keep a copy of this document for your records, please print or save this page now. You may also contact the researcher to request a copy.

By clicking the arrow below to be taken to the survey, you agree that you are over 18 years old, have read and

understood the above, and volunteer to participate in this study.

Part 1

How would you define the term “ultra-processed foods”?

Part 2

In the next section, you will be asked to select whether you think each food item is ultra-processed (“UPF”) or not ultra-processed (“Not UPF”). If you do not know, select “Don’t know/not sure.” You will also be asked to rate each food’s healthiness on a scale of 1-10, with 1 being the least healthy and 10 the most healthy. Please note the information provided below reflects information available on the package in most retail stores.

1

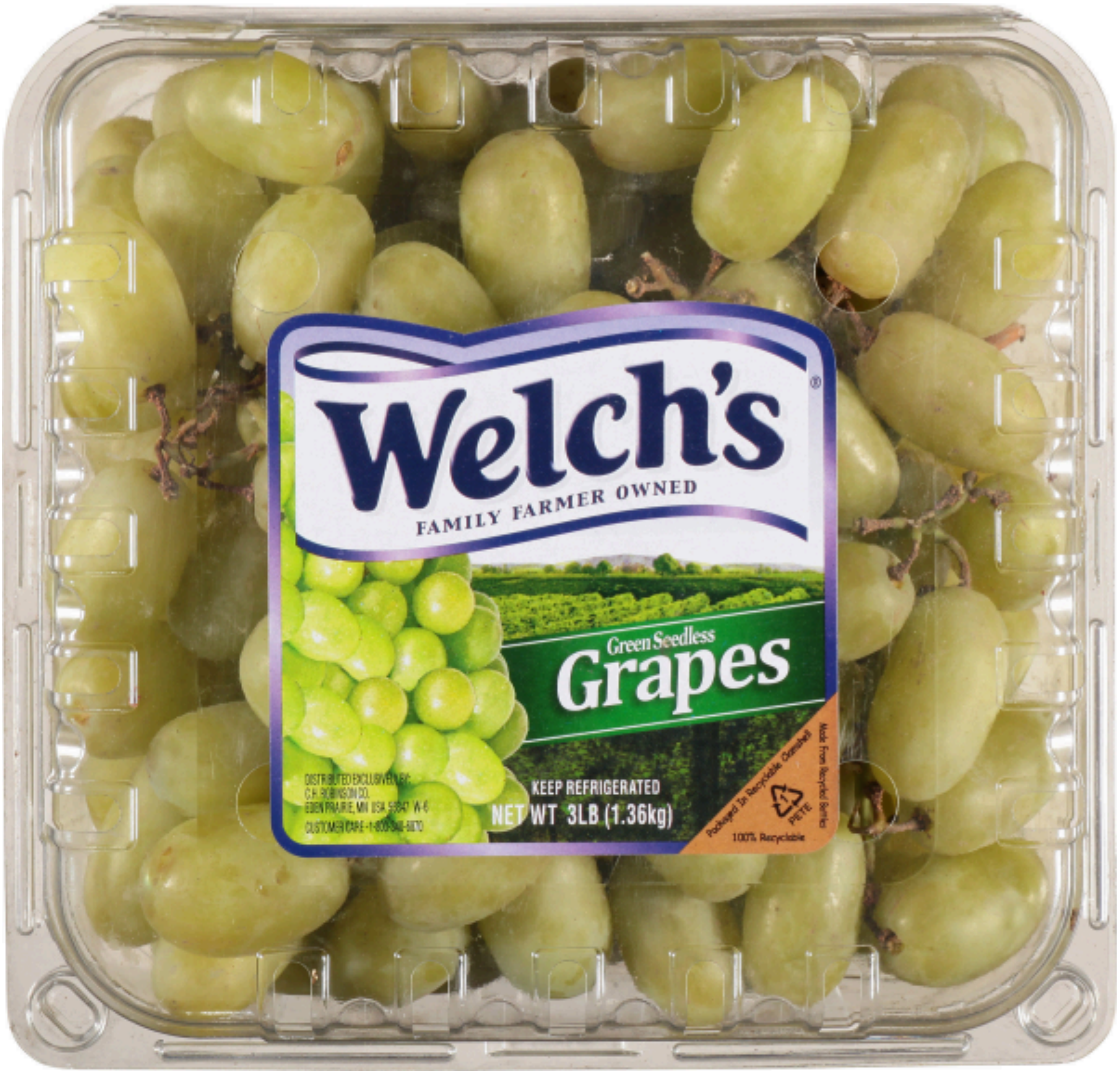

Processing Level

- ◯ UPF
- ◯ Not UPF
- ◯ Don't know/not sure

Healthiness

1	2	3	4	5	6	7	8	9	10
○	○	○	○	○	○	○	○	○	○

2

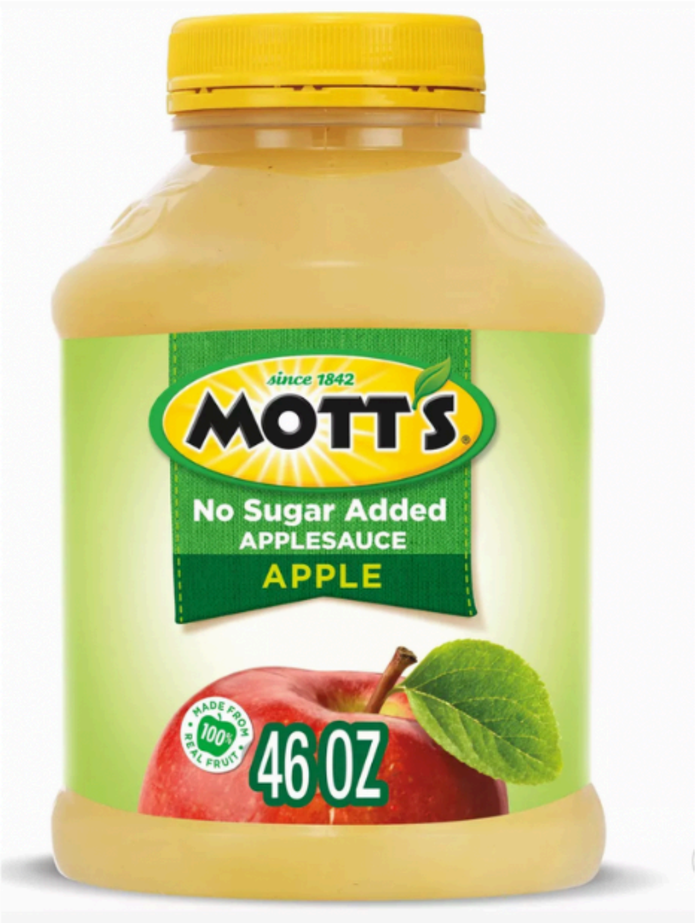

Nutrition Facts

About 11 servings per container

Serving size 1/2 cup (123g)

Amount per serving	
**Calories**	**50**
	**% Daily Value**
**Total Fat** 0g	**0%**
**Sodium** 0mg	**0%**
**Total Carbohydrate** 14g	**5%**
Dietary Fiber 1g	**4%**
Soluble Fiber 1g	
Total Sugars 12g	
Includes 0g Added Sugars	**0%**
**Protein** 0g	

Potassium 90mg 2% • Vitamin C 9mg 10%

Not a significant source of saturated fat, trans fat, cholesterol, vitamin D, calcium, and iron.

APPLES, WATER, ASCORBIC ACID (VITAMIN C).

Processing Level

- ○ UPF
- ○ Not UPF
- ○ Don't know/not sure

Healthiness

1 2 3 4 5 6 7 8 9 10

3

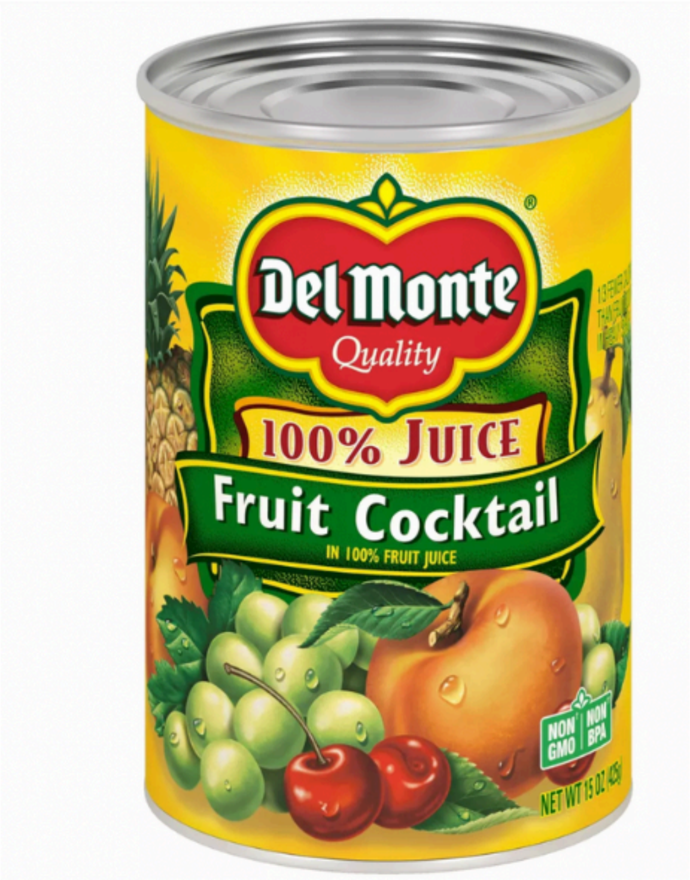

Nutrition Facts

Serving Size 124.00 g
Servings Per Container 3.5

Amount Per Serving	
**Calories** 60	
	**% Daily Value**
**Total Fat** 0g	**0%**
Saturated Fat 0g	**0%**
Trans Fat 0g	
**Cholesterol** 0mg	**0%**
**Sodium** 5mg	**0%**
**Total Carbohydrate** 15g	**5%**
Dietary Fiber 1g	**4%**
Sugars 12g	
**Protein** 1g	

Percent Daily Values are based on a 2,000 calorie diet.

Ingredients

Fruit (peaches, Pears, Grapes, Pineapple, Cherries [cherries, Carmine Color]), Peach Juice, Pear Juice, Natural Flavor, Ascorbic Acid (to Preserve Color)

Processing Level

- UPF
- Not UPF
- Don't know/not sure

Healthiness

1	2	3	4	5	6	7	8	9	10
○	○	○	○	○	○	○	○	○	○

4

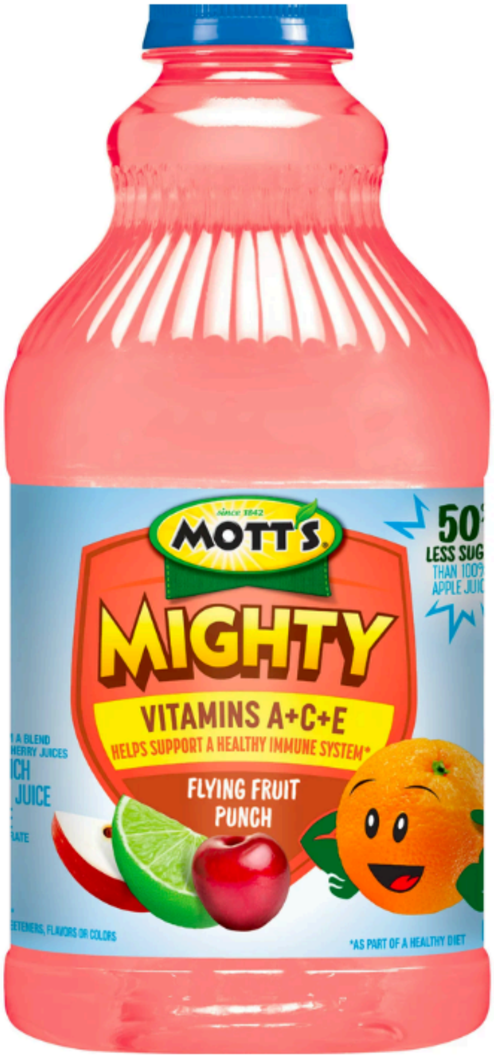

CONTAINS 49% JUICE

Nutrition Facts

8 servings per container

Serving size 8 fl oz (240mL)

Amount per serving

Calories 60

	% Daily Value
**Total Fat** 0g	**0%**
**Sodium** 5mg	**0%**
**Total Carbohydrate** 14g	**5%**
Total Sugars 13g	
Includes 0g Added Sugars	**0%**
**Protein** 0g	

Iron 0.4mg 2% • Potas. 140mg 4%

Vit. A 90mcg 10% • Vit. C 30mg 35%

Vitamin E 1.5mg 10%

Not a significant source of saturated fat, trans fat, cholesterol, dietary fiber, vitamin D, and calcium.

WATER, APPLE AND CHERRY JUICE CONCENTRATES, ASCORBIC ACID (VITAMIN C), NATURAL FLAVORS, VEGETABLE JUICE CONCENTRATE (COLOR), STEVIA LEAF EXTRACT, ALPHA TOCOPHERYL ACETATE (VITAMIN E), VITAMIN A PALMITATE.

Processing Level

- ○ UPF
- ○ Not UPF
- ○ Don't know/not sure

Healthiness

1 2 3 4 5 6 7 8 9 10

5

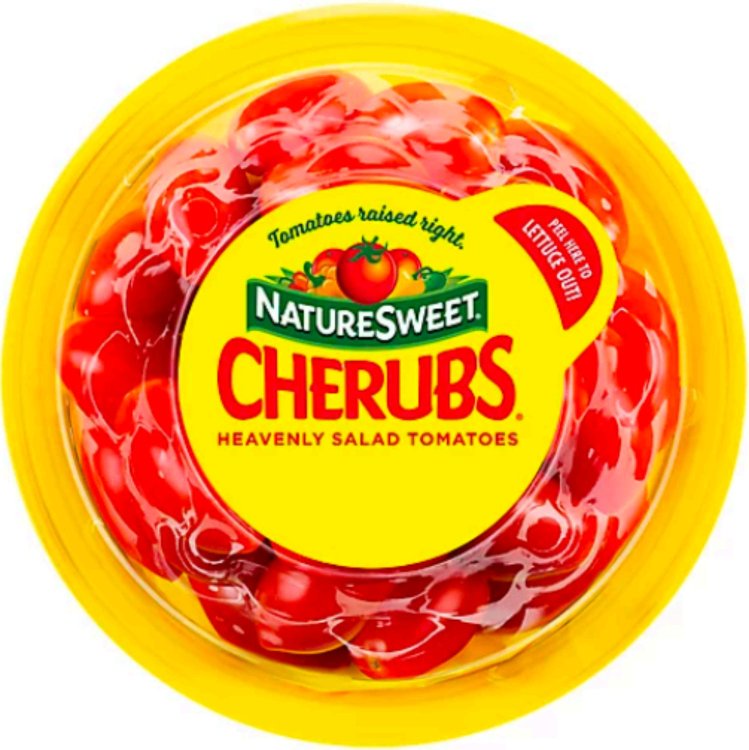

Processing Level

- UPF
- Not UPF
- Don't know/not sure

Healthiness

1	2	3	4	5	6	7	8	9	10
○	○	○	○	○	○	○	○	○	○

6

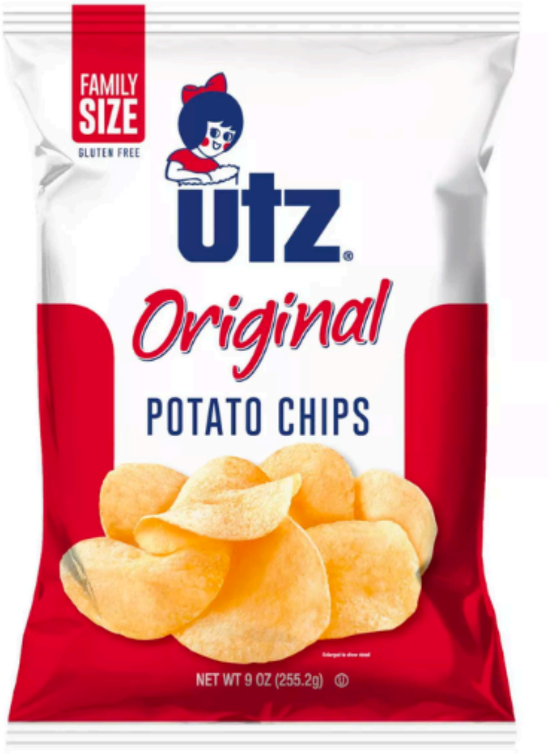

Nutrition Facts

9 servings per container
Serving size 1 oz (28g/about 20 chips)

Amount per serving	
**Calories**	**160**
	**% Daily Value***
**Total Fat** 10g	**13%**
Saturated Fat 2.5g	**13%**
Trans Fat 0g	
**Cholesterol** 0mg	**0%**
**Sodium** 95mg	**4%**
**Total Carbohydrate** 15g	**5%**
Dietary Fiber 1g	**4%**
Total Sugars 0g	
Includes 0g Added Sugars	**0%**
**Protein** 2g	
Vitamin D 0.1mcg	0%
Calcium 0mg	0%
Iron 0.4mg	2%
Potassium 390mg	8%

* The % Daily Value tells you how much a nutrient in a serving of food contributes to a daily diet. 2,000 calories a day is used for general nutrition advice.

INGREDIENTS: Potatoes, Cottonseed Oil, Salt.

Gluten Free

Processing Level

- ◯ UPF
- ◯ Not UPF
- ◯ Don't know/not sure

Healthiness

1 2 3 4 5 6 7 8 9 10

7

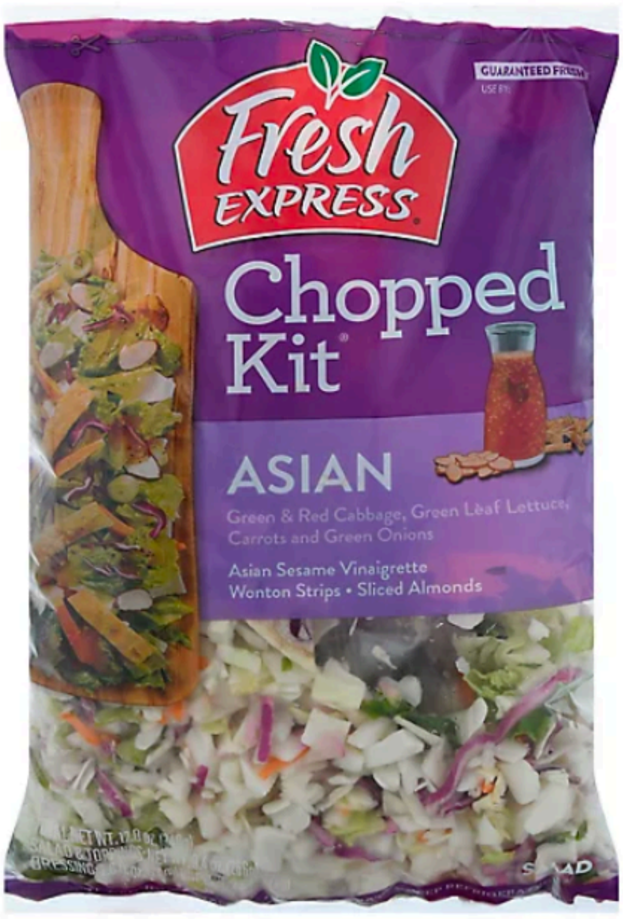

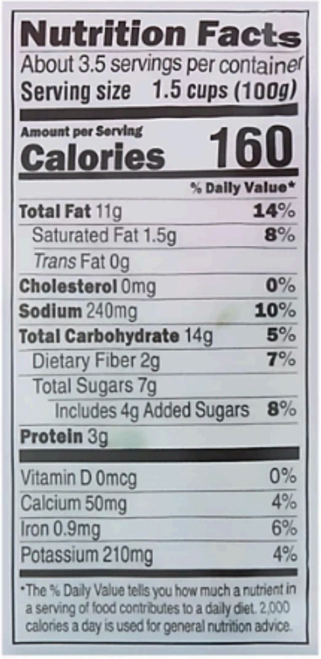

Nutrition Facts
About 3.5 servings per container
Serving size 1.5 cups (100g)

Amount per Serving	
**Calories**	**160**
	**% Daily Value***
**Total Fat** 11g	**14%**
Saturated Fat 1.5g	**8%**
Trans Fat 0g	
**Cholesterol** 0mg	**0%**
**Sodium** 240mg	**10%**
**Total Carbohydrate** 14g	**5%**
Dietary Fiber 2g	**7%**
Total Sugars 7g	
Includes 4g Added Sugars	**8%**
**Protein** 3g	
Vitamin D 0mcg	0%
Calcium 50mg	4%
Iron 0.9mg	6%
Potassium 210mg	4%

*The % Daily Value tells you how much a nutrient in a serving of food contributes to a daily diet. 2,000 calories a day is used for general nutrition advice.

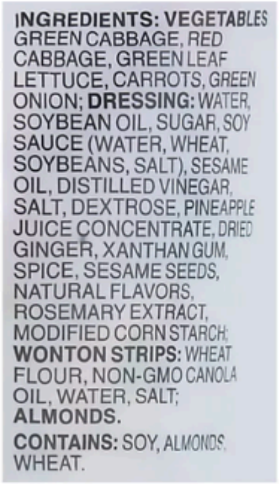

INGREDIENTS: VEGETABLES GREEN CABBAGE, RED CABBAGE, GREEN LEAF LETTUCE, CARROTS, GREEN ONION; **DRESSING:** WATER, SOYBEAN OIL, SUGAR, SOY SAUCE (WATER, WHEAT, SOYBEANS, SALT), SESAME OIL, DISTILLED VINEGAR, SALT, DEXTROSE, PINEAPPLE JUICE CONCENTRATE, DRIED GINGER, XANTHAN GUM, SPICE, SESAME SEEDS, NATURAL FLAVORS, ROSEMARY EXTRACT, MODIFIED CORN STARCH; **WONTON STRIPS:** WHEAT FLOUR, NON-GMO CANOLA OIL, WATER, SALT; **ALMONDS.**
CONTAINS: SOY, ALMONDS, WHEAT.

Processing Level

- UPF
- Not UPF
- Don't know/not sure

Healthiness

1	2	3	4	5	6	7	8	9	10
○	○	○	○	○	○	○	○	○	○

8

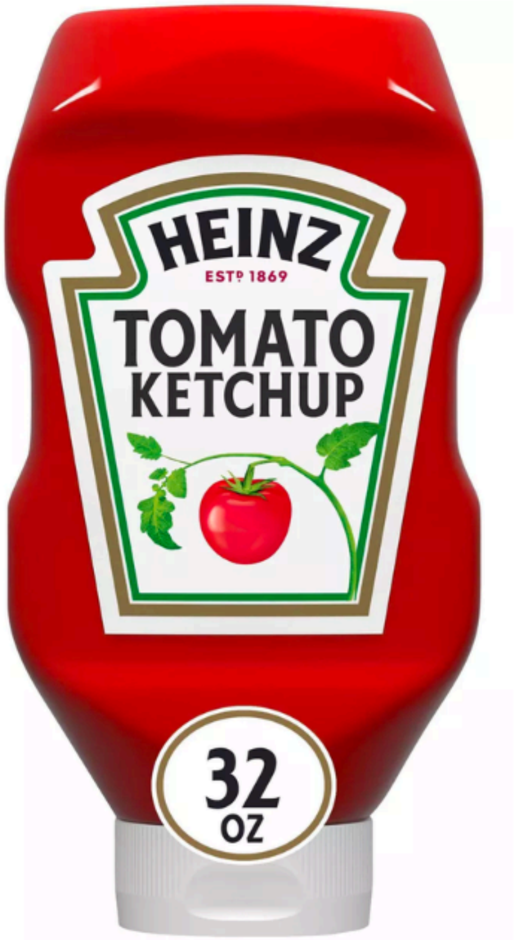

20	0g	180mg	4g
CALORIES	SAT FAT	SODIUM	SUGARS

Nutrition Facts

About 23 servings per container

Serving Size **1.0 tbsp**

Amount per serving

Calories **20**

	% Daily Value*
**Total Fat** 0g	0%
Saturated Fat 0g	0%
Trans Fat 0g	
**Cholesterol** 0mg	0 %
**Sodium** 180mg	8 %
**Total Carbohydrate** 5g	2 %
Dietary Fiber 0g	0 %
Total Sugars 4g	
Includes 4g Added Sugars	7 %
**Protein** 0g	
Vitamin D	0 %
Calcium	0 %
Iron	0 %
Potassium	0%

*The % Daily Value (DV) tells you how much a nutrient in a serving of food contributes to a daily diet. 2,000 calories a day is used for general nutrition advice.

Ingredients
Tomato Concentrate From Red Ripe Tomatoes, Distilled Vinegar, High Fructose Corn Syrup, Corn Syrup, Salt, Spice, Onion Powder, Natural Flavoring.

Processing Level

- ○ UPF
- ○ Not UPF
- ○ Don't know/not sure

Healthiness

○ 1 ○ 2 ○ 3 ○ 4 ○ 5 ○ 6 ○ 7 ○ 8 ○ 9 ○ 10

9

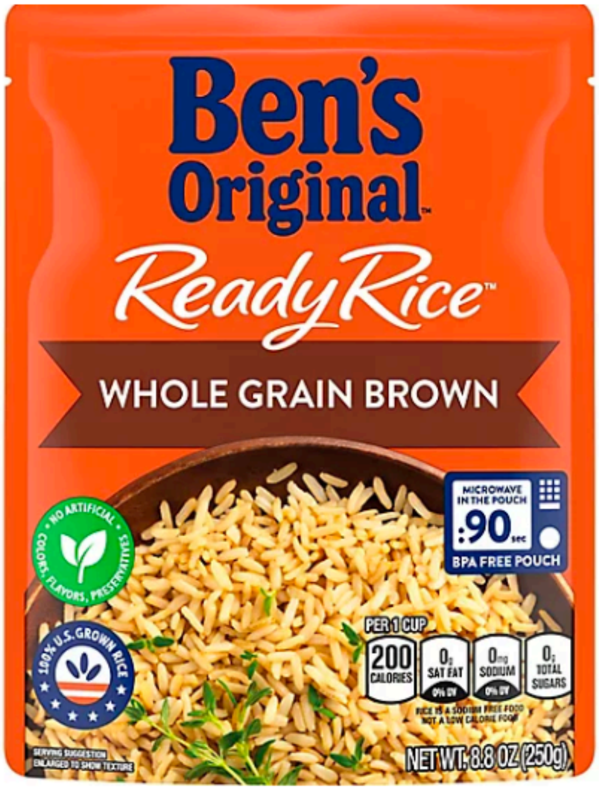

Nutrition Facts

Serving size 1 Pouch (250g)	Per Cup (140g)		Per pouch	
**Calories**	**200**		**360**	
		**% DV***		**% DV***
**Total Fat**	3g	**4%**	5g	**6%**
Saturated Fat	0g	**0%**	1g	**5%**
Trans Fat	0g		0g	
Polyunsaturated fat	1g		1.5g	
Monounsaturated Fat	1.5g		3g	
**Cholesterol**	0mg	**0%**	0mg	**0%**
**Sodium**	0mg	**0%**	5mg	**0%**
**Total Carbohydrate**	40g	**15%**	71g	**26%**
Dietary Fiber	<1g	**4%**	1g	**4%**
Total Sugars	0g		<1g	
Incl. Added Sugars	0g	**0%**	0g	**0%**
**Protein**	4g		7g	
Vitamin D	0mcg	0%	0mcg	0%
Calcium	80mg	6%	150mg	10%
Iron	0.4mg	2%	0.8mg	4%
Potassium	70mg	0%	130mg	2%

* The % Daily Value (DV) tells you how much a nutrient in a serving of food contributes to a daily diet. 2,000 calories a day is used for general nutrition advice.

INGREDIENTS: WATER, PARBOILED LONG GRAIN BROWN RICE; LESS THAN 2% OF: CANOLA OIL†.
† ADDS A TRIVIAL AMOUNT OF SATURATED FAT.
CONTAINS A BIOENGINEERED FOOD INGREDIENT.

Processing Level

- UPF
- Not UPF
- Don't know/not sure

Healthiness

1	2	3	4	5	6	7	8	9	10
○	○	○	○	○	○	○	○	○	○

10

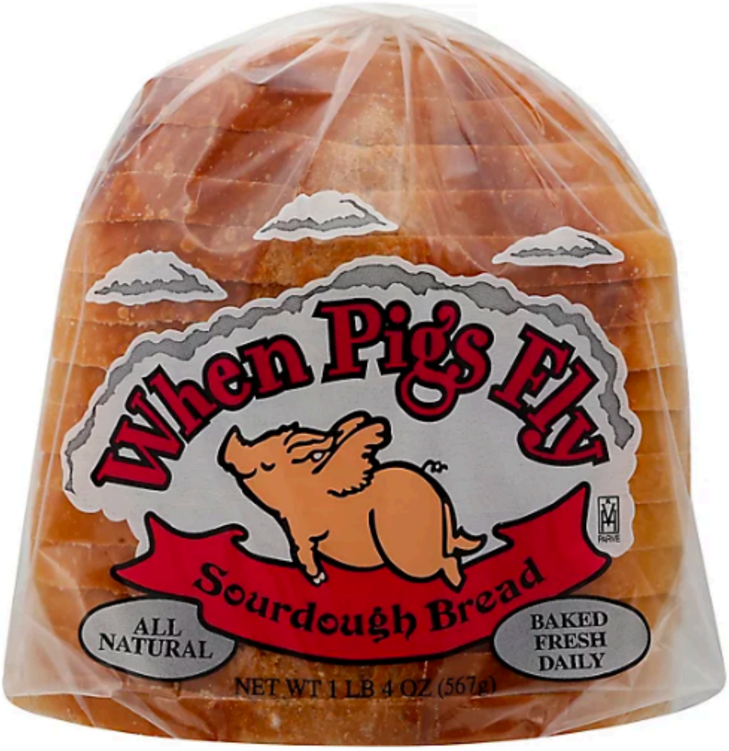

Nutrition Facts

Serving Size: 1 average slice (48g)
Servings Per Container: approx. 13

Amount Per Serving	
**Calories** 110	Calories from Fat 5
	**% Daily Value***
**Total Fat** 0g	**0%**
Saturated Fat 0g	**0%**
Trans Fat 0g	
**Cholesterol** 0mg	**0%**
**Sodium** 170mg	**7%**
**Total Carbohydrate** 23g	**8%**
Dietary Fiber 1g	**4%**
Sugars 1g	
**Protein** 3g	
Vitamin A 0% • Vitamin C 0%	
Calcium 0% • Iron 6%	

*Percent Daily Values are based on a 2,000 calorie diet.

INGREDIENTS: UNBLEACHED FLOUR, CIDER, EVAPORATED CANE JUICE, SALT AND YEAST.

Processing Level

- ○ UPF
- ○ Not UPF
- ○ Don't know/not sure

Healthiness

○ 1 ○ 2 ○ 3 ○ 4 ○ 5 ○ 6 ○ 7 ○ 8 ○ 9 ○ 10

11

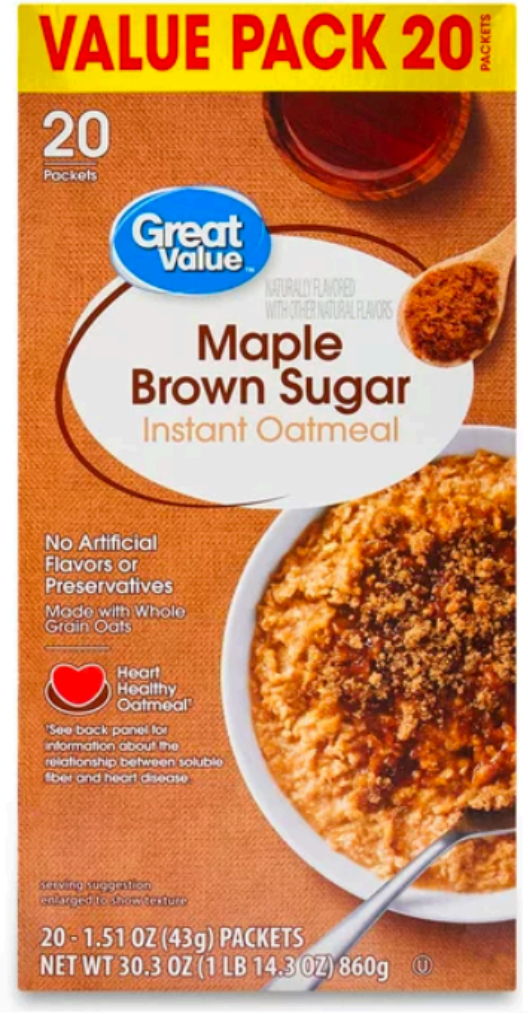

Nutrition Facts

20 servings per container
Serving size 1 packet (43g)

Amount per serving
Calories 160

	% Daily Value*
**Total Fat** 2g	**3%**
Saturated Fat 0g	**0%**
Trans Fat 0g	
Polyunsaturated Fat 1g	
Monounsaturated Fat 1g	
**Cholesterol** 0mg	**0%**
**Sodium** 260mg	**11%**
**Total Carbohydrate** 32g	**12%**
Dietary Fiber 3g	**10%**
Soluble Fiber 1g	
Total Sugars 12g	
Includes 12g Added Sugars	**23%**
**Protein** 4g	
Vitamin D 0mcg	0%
Calcium 160mg	10%
Iron 3.6mg	20%
Potassium 110mg	2%
Vitamin A	20%
Thiamine	20%
Riboflavin	20%
Niacin	20%
Vitamin B_6	20%
Folate 80mcg DFE (50mcg folic acid)	20%
Phosphorus	10%
Magnesium	10%

* The % Daily Value tells you how much a nutrient in a serving of food contributes to a daily diet. 2,000 calories a day is used for general nutrition advice.

Calories per gram:
Fat 9 • Carbohydrate 4 • Protein 4

INGREDIENTS: WHOLE GRAIN ROLLED OATS, SUGAR, CONTAINS LESS THAN 2% OF SALT, GUAR GUM, NATURAL FLAVOR, CARAMEL COLOR.

VITAMINS AND MINERALS: CALCIUM CARBONATE (A SOURCE OF CALCIUM), FERRIC ORTHOPHOSPHATE (A SOURCE OF IRON), VITAMIN A PALMITATE, NIACINAMIDE, PYRIDOXINE HYDROCHLORIDE (VITAMIN B_6), RIBOFLAVIN (VITAMIN B_2), THIAMINE MONONITRATE (VITAMIN B_1), FOLIC ACID.

Processing Level

- UPF
- Not UPF
- Don't know/not sure

Healthiness

1	2	3	4	5	6	7	8	9	10
○	○	○	○	○	○	○	○	○	○

12

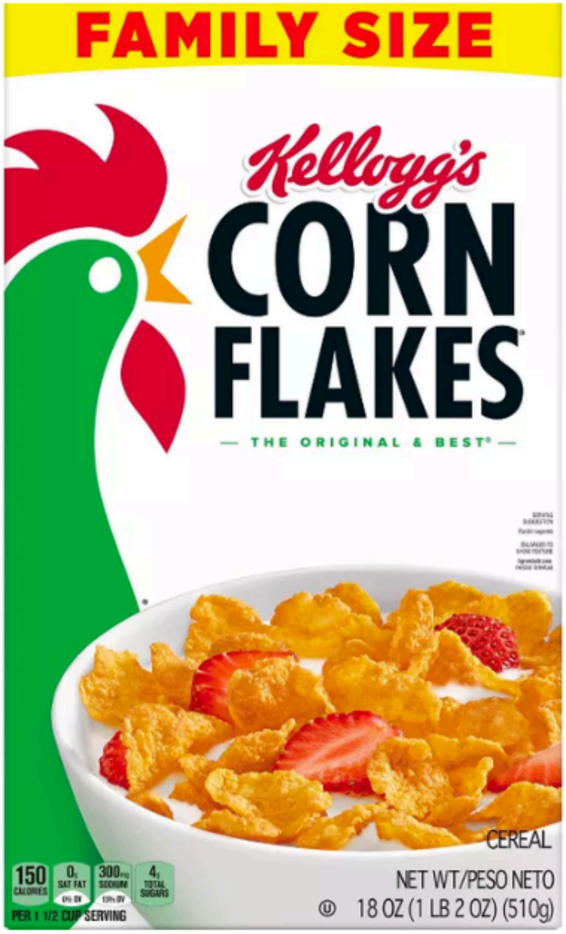

150	0 g	300 mg	4 g
CALORIES	SAT FAT	SODIUM	SUGARS

Nutrition Facts

about 6 servings per container

Serving Size **1.5 cup**

Amount per serving

Calories **150**

	**% Daily Value***
**Total Fat** 0g	**0%**
Saturated Fat 0g	**0%**
Trans Fat 0g	
Polyunsaturated Fat 0g	
Monounsaturated Fat 0g	
**Cholesterol** 0mg	**0 %**
**Sodium** 300mg	**13 %**
**Total Carbohydrate** 36g	**13 %**
Dietary Fiber 1g	**5 %**
Total Sugars 4g	
Includes 4g Added Sugars	**8 %**
**Protein** 3g	
Vitamin D 3mcg	**15 %**
Calcium 0mg	**0 %**
Iron 12mg	**60 %**
Potassium 60mg	**0 %**

*The % Daily Value (DV) tells you how much a nutrient in a serving of food contributes to a daily diet. 2,000 calories a day is used for general nutrition advice.

Ingredients

Milled Corn, Sugar, Malt Flavor, Contains 2% Or Less Of Salt, Vitamins And Minerals: Iron (Ferric Phosphate), Niacinamide, Vitamin B6 (Pyridoxine Hydrochloride), Vitamin B2 (Riboflavin), Vitamin B1 (Thiamin Hydrochloride), Folic Acid, Vitamin D3, Vitamin B12.

Processing Level

- ○ UPF
- ○ Not UPF
- ○ Don't know/not sure

Healthiness

○ 1 ○ 2 ○ 3 ○ 4 ○ 5 ○ 6 ○ 7 ○ 8 ○ 9 ○ 10

13

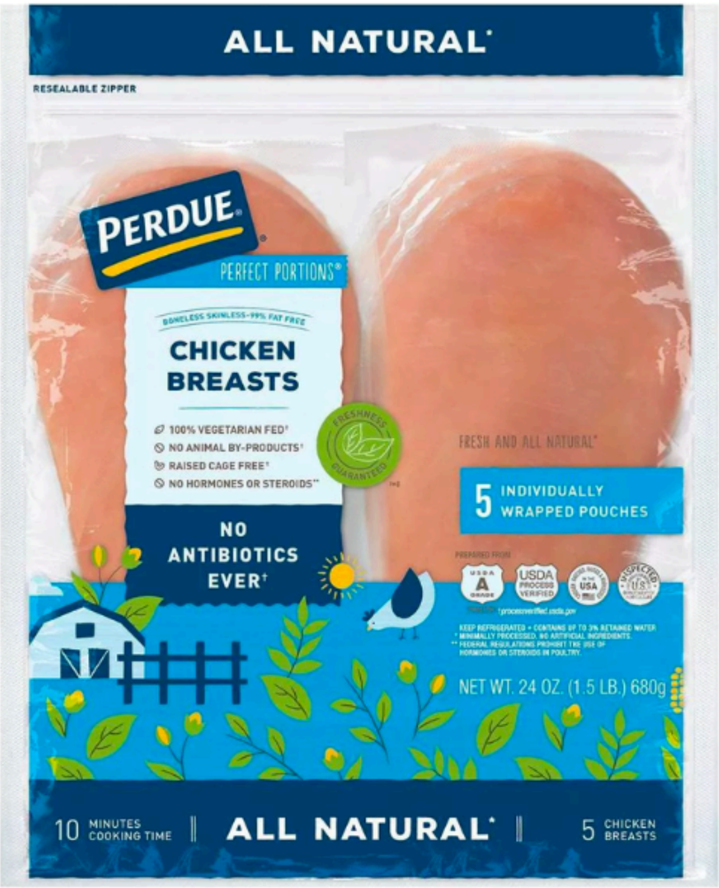

130	0 g	90 mg	0 g
CALORIES	SAT FAT	SODIUM	SUGARS

Nutrition Facts

5 servings per container

**Serving Size**	**1.0 filet**
**Amount per serving**	
**Calories**	**130**
	**% Daily Value***
**Total Fat** 1.5g	**2%**
Saturated Fat 0g	**0%**
Trans Fat 0g	
**Cholesterol** 100mg	**33 %**
**Sodium** 90mg	**4 %**
**Total Carbohydrate** 0g	**0 %**
Dietary Fiber 0g	**0 %**
Total Sugars 0g	
**Protein** 31g	
Vitamin A	**0 %**
Vitamin C	**0 %**
Calcium	**0 %**
Iron	**2 %**

*The % Daily Value (DV) tells you how much a nutrient in a serving of food contributes to a daily diet. 2,000 calories a day is used for general nutrition advice.

Ingredients

Boneless Skinless Chicken Breast.

Processing Level

- UPF
- Not UPF
- Don't know/not sure

Healthiness

1	2	3	4	5	6	7	8	9	10
○	○	○	○	○	○	○	○	○	○

14

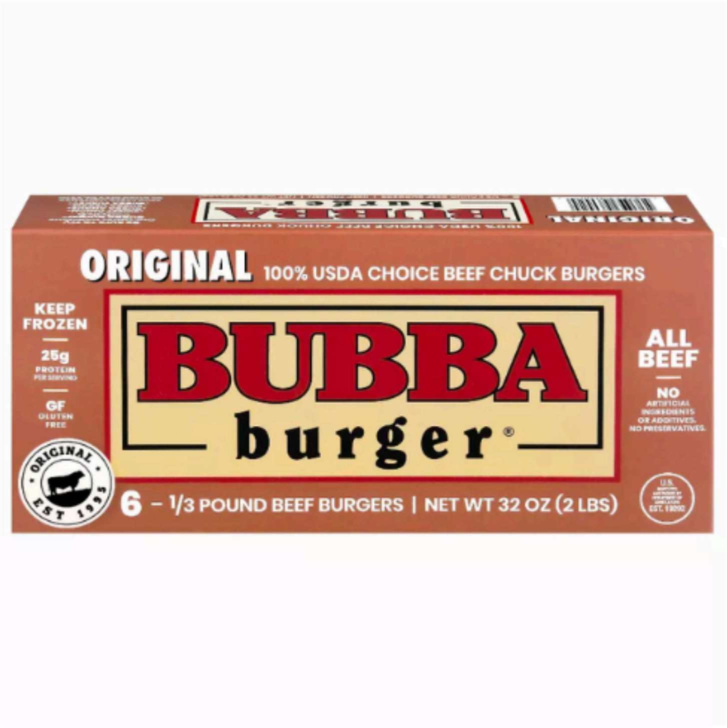

Nutrition Facts

Serving Size: 1 Burger (151g)
Servings Per Container: 6

Amount Per Serving	
**Calories** 420	Calories from fat 320
	**% Daily Value***
**Total Fat** 35g	**54%**
Saturated Fat 15g	**75%**
**Cholesterol** 110mg	**37%**
**Sodium** 85mg	**4%**
**Total Carbohydrate** 0g	**0%**
Dietary Fiber 0g	**0%**
Sugars 0g	
**Protein** 25g	

Vitamin A	0%	Vitamin C	0%
Calcium	0%	Iron	15%

* Percent Daily Values are based on a 2,000 calorie diet. Your daily values may be higher or lower depending on your calorie needs.

	Calories	2,000	2,500
Total Fat	Less than	65g	80g
Saturated Fat	Less than	20g	25g
Cholesterol	Less than	300mg	300mg
Sodium	Less than	2,400mg	2,400mg
Total Carbohydrate		300g	375g
Dietary Fiber		25g	30g

Calories per gram
Fat 9 • Carbohydrate 4 • Protein 4

INGREDIENTS: USDA CHOICE BEEF CHUCK

Processing Level

- UPF
- Not UPF
- Don't know/not sure

Healthiness

1 2 3 4 5 6 7 8 9 10

15

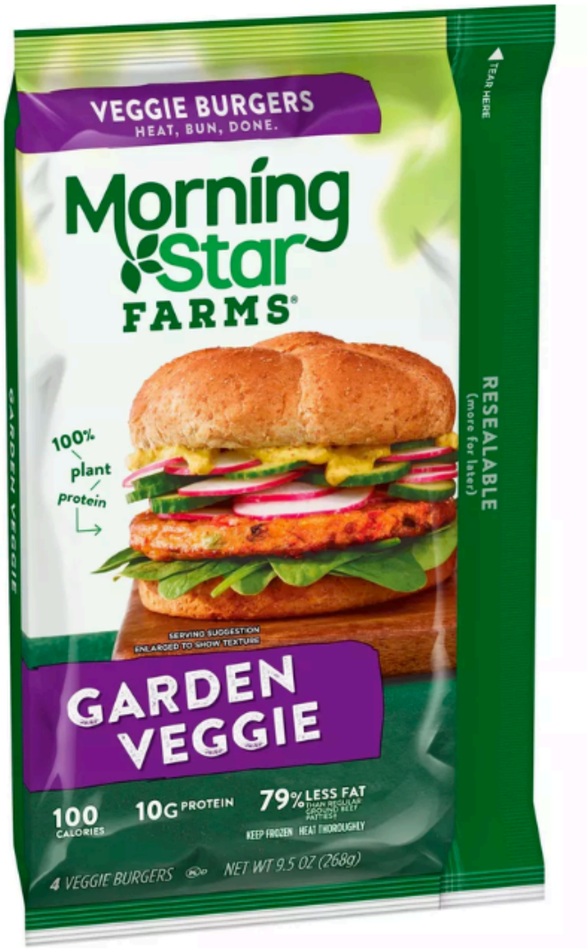

Nutrition Facts

4 servings per container
Serving size 1 Burger (67g)

Amount per serving	
**Calories**	**110**
	**% Daily Value***
**Total Fat** 4.5g	**6%**
Saturated Fat 0.5g	**3%**
Trans Fat 0g	
**Cholesterol** 0mg	**0%**
**Sodium** 390mg	**17%**
**Total Carbohydrate** 9g	**3%**
Dietary Fiber 4g	**14%**
Total Sugars 1g	
Includes 0g Added Sugars	**0%**
**Protein** 11g	**17%**
Vitamin D 0.1mcg 0% • Calcium 60mg 4%	
Iron 0.9mg 4% • Potassium 150mg 2%	

* The % Daily Value (DV) tells you how much a nutrient in a serving of food contributes to a daily diet. 2,000 calories a day is used for general nutrition advice.

Ingredients: Water, carrots, onions, soy flour, egg whites, mushrooms, whole grain oats, wheat gluten, water chestnuts, vegetable oil (corn, canola, and/or sunflower oil), **green bell peppers, calcium caseinate** (from milk), **cooked brown rice** (water, brown rice), **red bell peppers.**

Contains 2% or less of onion powder, cornstarch, soy sauce powder (soy sauce [soybeans, salt, wheat]), sugar, black olives, salt, spices, garlic powder, jalapeno peppers, xanthan gum.

CONTAINS SOY, EGG, WHEAT AND MILK INGREDIENTS.

Processing Level

- UPF
- Not UPF
- Don't know/not sure

Healthiness

1	2	3	4	5	6	7	8	9	10
○	○	○	○	○	○	○	○	○	○

16

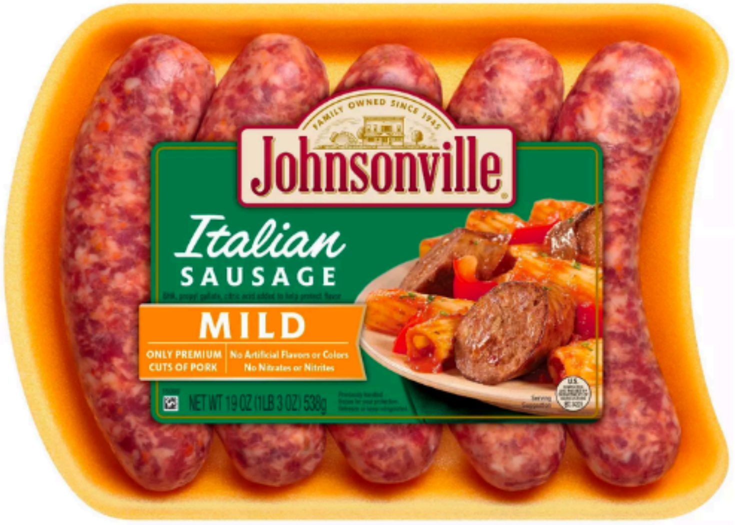

170	4.5 g	450 mg	1 g
CALORIES	SAT FAT	SODIUM	SUGARS

Nutrition Facts

about 6 servings per container
Serving Size **2.0 oz (cooked portion)**

Amount per serving
Calories **170**

	**% Daily Value***
**Total Fat** 13g	**21%**
Saturated Fat 4.5g	**23%**
Trans Fat 0g	
**Cholesterol** 40mg	**13 %**
**Sodium** 450mg	**19 %**
**Total Carbohydrate** 1g	**0 %**
Dietary Fiber 0g	**0 %**
Total Sugars 1g	
**Protein** 10g	
Vitamin A	**2 %**
Vitamin C	**0 %**
Calcium	**2 %**
Iron	**2 %**

*The % Daily Value (DV) tells you how much a nutrient in a serving of food contributes to a daily diet. 2,000 calories a day is used for general nutrition advice.

Ingredients
Pork, Water, And Less Than 2% Of The Following: Pork Broth With Natural Flavorings, Salt, Natural Sugar, Spices, Paprika, Natural Flavors.

Processing Level

- ○ UPF
- ○ Not UPF
- ○ Don't know/not sure

Healthiness

○ 1 ○ 2 ○ 3 ○ 4 ○ 5 ○ 6 ○ 7 ○ 8 ○ 9 ○ 10

17

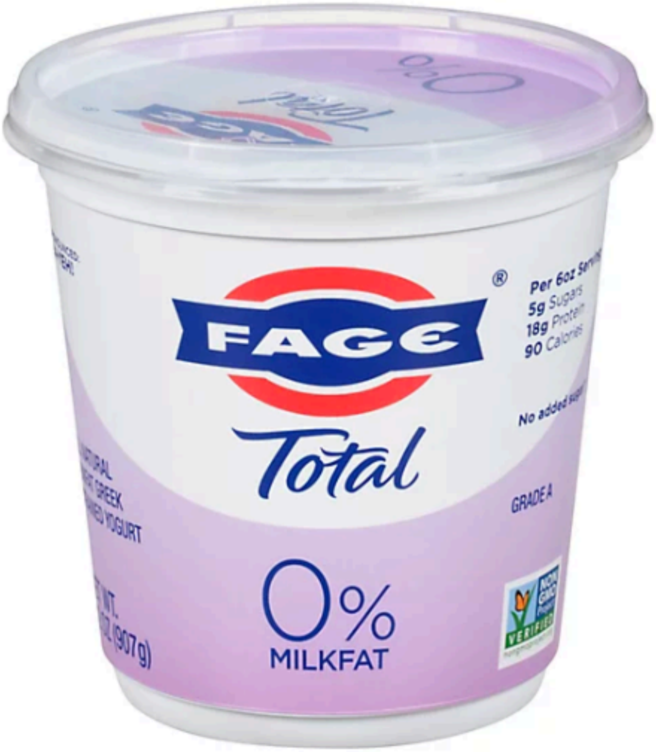

Nutrition Facts

About 5 servings per container

Serving Size	3/4 cup (170g)
Amount per serving **Calories**	**90**
	% Daily Value*
**Total Fat** 0g	0%
Saturated Fat 0g	0%
Trans Fat 0g	
**Cholesterol** 10mg	3%
**Sodium** 65mg	3%
**Total Carbohydrate** 5g	2%
Dietary Fiber 0g	0%
Total Sugars 5g	
Includes 0g Added Sugars	0%
**Protein** 18g	35%
Vitamin D 0mcg 0% • Calcium 200mg 15%	
Iron 0mg 0% • Potassium 260mg 6%	

*The % Daily Value tells you how much a nutrient in a serving of food contributes to a daily diet. 2000 calories a day is used for general nutrition advice.

INGREDIENTS: GRADE A PASTEURIZED SKIMMED MILK, LIVE ACTIVE YOGURT CULTURES (L. BULGARICUS, S. THERMOPHILUS, L. ACIDOPHILUS, BIFIDUS, L. CASEI).

Processing Level

- UPF
- Not UPF
- Don't know/not sure

Healthiness

1	2	3	4	5	6	7	8	9	10
○	○	○	○	○	○	○	○	○	○

18

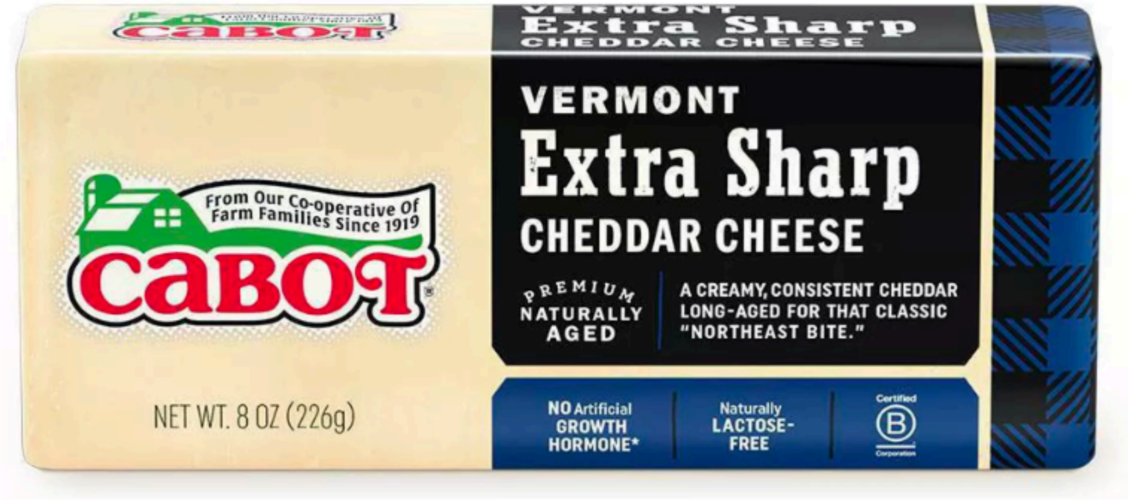

Nutrition Facts

8 servings
Serving size
1oz (28g/1" Cube)

Calories per serving 110

Amount/serving	% DV*	Amount/serving	% DV*
Total Fat 9g	12%	Total Carb. 1g	0%
Sat. Fat 6g	30%	Fiber 0g	0%
Trans. Fat 0g		Total Sugars 0g	
Cholesterol 30mg	10%	Includes 0g Added Sugars	0%
Sodium 200mg	9%	Protein 7g	

Vit D 0mcg 0% · Calcium 194mg 15% · Iron 0mg 0% · Potas 20mg 0%

* The % Daily Value (DV) tells you how much a nutrient in a serving of food contributes to a daily diet. 2,000 calories a day is used for general nutrition advice.

INGREDIENTS: Fresh pasteurized milk, cheese cultures, salt, enzymes.

CONTAINS NO ANIMAL RENNET.
CONTAINS 0g OF LACTOSE PER SERVING.

Processing Level

- ◯ UPF
- ◯ Not UPF
- ◯ Don't know/not sure

Healthiness

○ 1 ○ 2 ○ 3 ○ 4 ○ 5 ○ 6 ○ 7 ○ 8 ○ 9 ○ 10

19

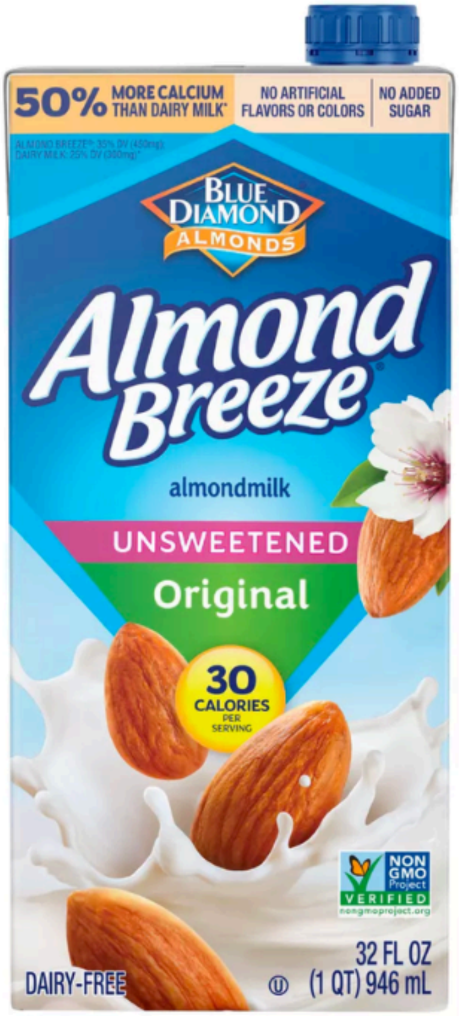

Nutrition Facts

4 servings per container

Serving size **1 cup (240mL)**

Amount per serving	
**Calories**	**30**
	**% Daily Value***
**Total Fat** 2.5g	**3%**
Saturated Fat 0g	**0%**
Trans Fat 0g	
Polyunsaturated Fat 0.5g	
Monounsaturated Fat 1.5g	
**Cholesterol** 0mg	**0%**
**Sodium** 170mg	**7%**
**Total Carbohydrate** 1g	**0%**
Dietary Fiber <1g	**2%**
Total Sugars 0g	
Includes 0g Added Sugars	**0%**
**Protein** 1g	
Vitamin D 0mcg	0%
Calcium 450mg	35%
Iron 0.7mg	4%
Potassium 160mg	4%
Vitamin E 5mg	35%
Phosphorus 20mg	2%
Magnesium 15mg	4%

*The % Daily Value (DV) tells you how much a nutrient in a serving of food contributes to a daily diet. 2,000 calories a day is used for general nutrition advice.

INGREDIENTS: ALMONDMILK (FILTERED WATER, ALMONDS), CALCIUM CARBONATE, SEA SALT, POTASSIUM CITRATE, SUNFLOWER LECITHIN, GELLAN GUM, NATURAL FLAVORS, D-ALPHA-TOCOPHEROL (NATURAL VITAMIN E).

CONTAINS: ALMONDS.

Processing Level

- UPF
- Not UPF
- Don't know/not sure

Healthiness

1	2	3	4	5	6	7	8	9	10
○	○	○	○	○	○	○	○	○	○

20

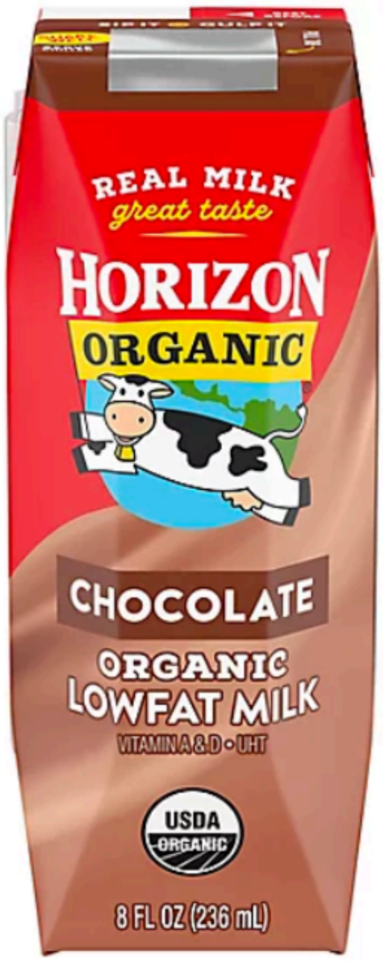

Nutrition Facts

Serv size 1 Milk Box (236mL)

Amount per serving
Calories 150

	% Daily Value*
**Total Fat** 2.5g	**3%**
Sat Fat 1.5g	**8%**
Trans Fat 0g	
**Cholest** 15mg	**5%**
**Sodium** 180mg	**8%**
**Total Carb** 23g	**8%**
Fiber 0g	**0%**
Total Sugars 22g	
Incl. 10g Added Sugars	**20%**
**Protein** 8g	**16%**

Vit D 2.5mcg 15% • Calcium 290mg 20%
Iron 0.6mg 4% • Potassium 450mg 10%
Vit A 150mcg 15% • Riboflavin 0.4mg 30%

*The % Daily Value (DV) tells you how much a nutrient in a serving of food contributes to a daily diet. 2,000 calories a day is used for general nutrition advice.

INGREDIENTS:
Grade A Lowfat Organic Milk, Organic Cane Sugar, Organic Cocoa (Processed with Alkali), Organic Cocoa, Gellan Gum, Organic Natural Flavor, Salt, Vitamin A Palmitate, Vitamin D3.
CONTAINS MILK.

Processing Level

- ○ UPF
- ○ Not UPF
- ○ Don't know/not sure

Healthiness

1 2 3 4 5 6 7 8 9 10

21

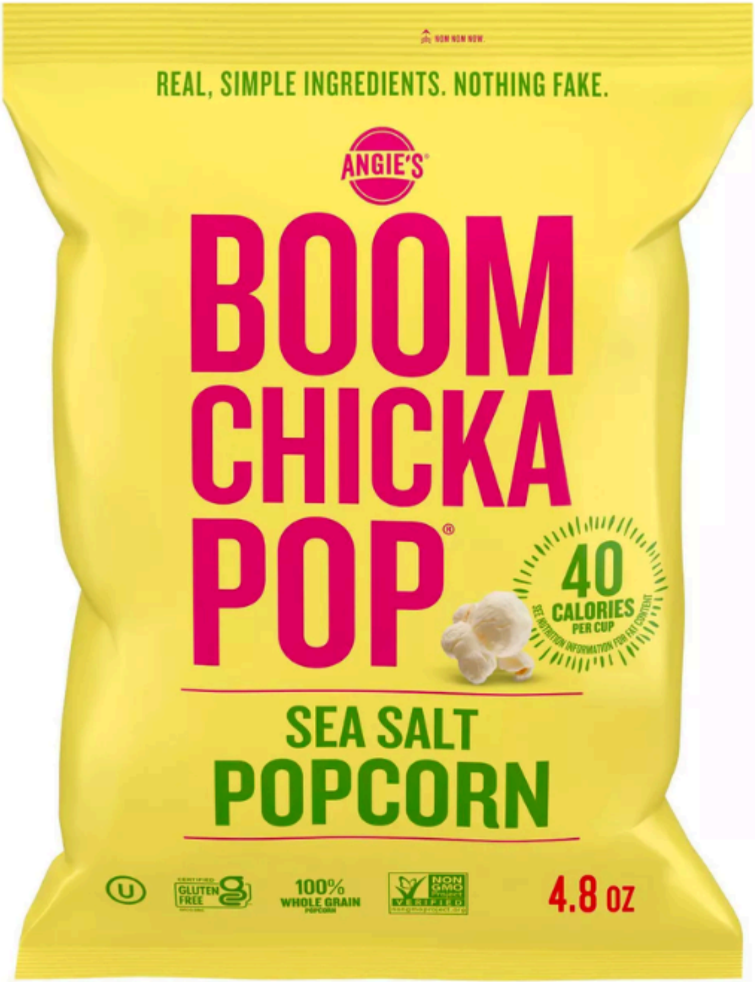

Nutrition Facts

about 5 servings per container

Serving size 3 ¾ cups (28g)

	Per serving		Per 1 cup	
**Calories**	**150**		**40**	
		% DV*		% DV*
**Total Fat**	9g	**12%**	2.5g	**3%**
Saturated Fat	1g	**5%**	0g	**0%**
Trans Fat	0g		0g	
Polyunsaturated Fat	2g		0.5g	
Monounsaturated Fat	5g		1.5g	
**Cholesterol**	0mg	**0%**	0mg	**0%**
**Sodium**	150mg	**7%**	40mg	**2%**
**Total Carb.**	16g	**6%**	4g	**1%**
Dietary Fiber	2g	**7%**	less than 1g	**2%**
Total Sugars	0g		0g	
Incl. Added Sugars	0g	**0%**	0g	**0%**
**Protein**	2g		less than 1g	
Vitamin D	0mcg	0%	0mcg	0%
Calcium	0mg	0%	0mg	0%
Iron	0.4mg	2%	0mg	0%
Potassium	0mg	0%	0mg	0%

*The % Daily Value (DV) tells you how much a nutrient in a serving of food contributes to a daily diet. 2,000 calories a day is used for general nutrition advice.

INGREDIENTS: POPCORN, SUNFLOWER OIL, SEA SALT.

Processing Level

- UPF
- Not UPF
- Don't know/not sure

Healthiness

1	2	3	4	5	6	7	8	9	10
○	○	○	○	○	○	○	○	○	○

22

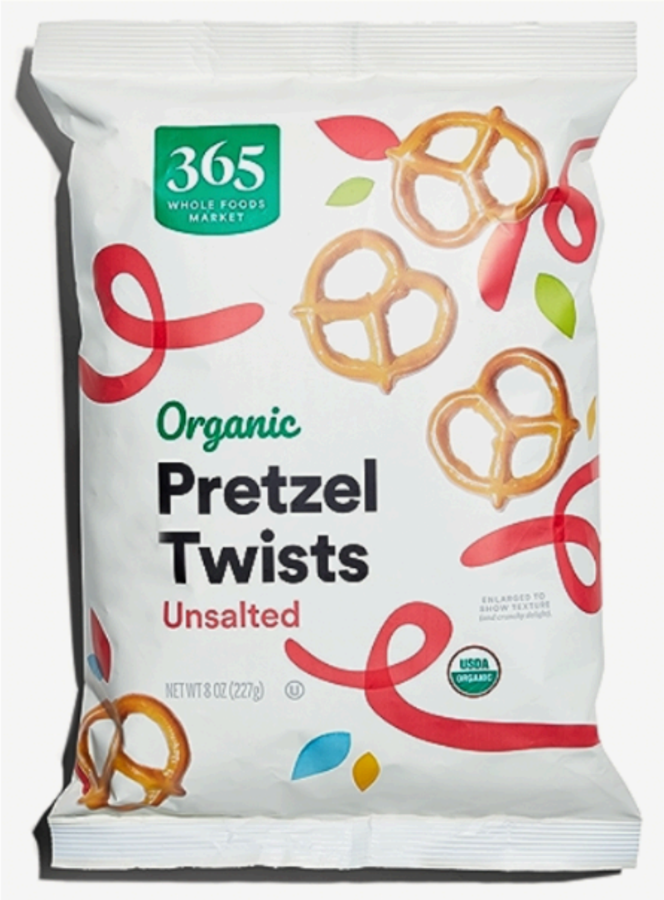

Nutrition Facts

8 servings per container

**Serving size**	**1 oz (28 g)**
**Amount per serving**	
**Calories**	**110**
	**% Daily Value ***
**Total Fat** 0.5g	**0.64%**
Sat Fat 0g	**0%**
Trans Fat 0g	
Polyunsaturated Fat 0g	
Monounsaturated Fat 0g	
**Cholesterol** 0mg	**0%**
**Sodium** 40mg	**1.7%**
**Carbohydrates** 23g	**8%**
Fiber 2g	**7%**
Sugars < 1g	
Added Sugar 0g	**0%**
**Protein** 3g	
Vitamin D 0mcg	0%
Potassium 0mg	0%
Iron 0.4mg	2%
Calcium 0mg	0%

* The % Daily Value (DV) tells you how much a nutrient in a serving of food contributes to a daily diet. 2,000 calories a day is used for general nutrition advice.

Ingredients: Organic Wheat Flour, Organic Expeller Pressed Soybean Oil, Organic Malted Barley Flour.

Processing Level

- ○ UPF
- ○ Not UPF
- ○ Don't know/not sure

Healthiness

1 2 3 4 5 6 7 8 9 10

23

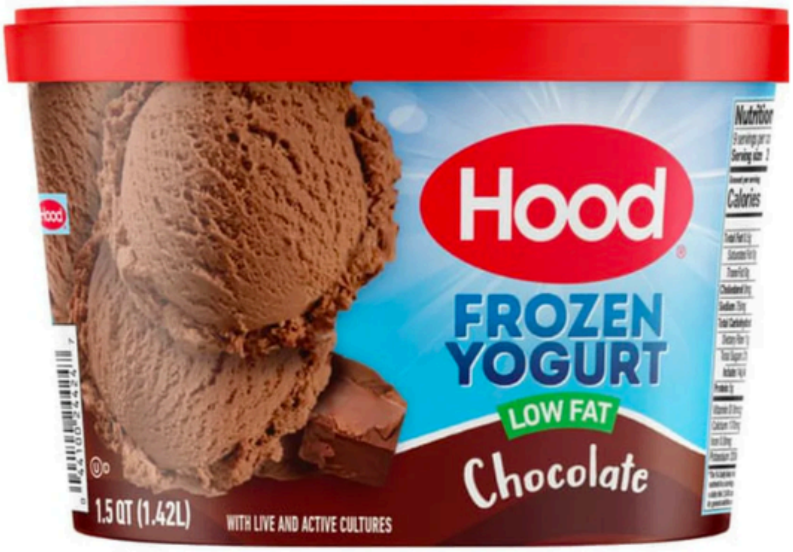

INGREDIENTS: NONFAT MILK, SUGAR, CULTURED NONFAT MILK, CORN SYRUP, COCOA PROCESSED WITH ALKALI, MONO & DIGLYCERIDES, LOCUST BEAN GUM, CELLULOSE GUM, GUAR GUM AND CARRAGEENAN.

CONTAINS: MILK.

Nutrition Facts

9 servings per container

Serving size 2/3 cup (87g)

Amount per serving	
**Calories**	**130**
	**% Daily Value***
**Total Fat** 0.5g	**1%**
Saturated Fat 0g	**0%**
Trans Fat 0g	
**Cholesterol** 0mg	**0%**
**Sodium** 75mg	**3%**
**Total Carbohydrate** 26g	**9%**
Dietary Fiber 1g	**4%**
Total Sugars 21g	
Includes 14g Added Sugars	**28%**
**Protein** 5g	
Vitamin D 0mcg	0%
Calcium 170mg	15%
Iron 0.8mg	4%
Potassium 320mg	6%

*The % Daily Value tells you how much a nutrient in a serving of food contributes to a daily diet. 2,000 calories a day is used for general nutrition advice.

Processing Level

- UPF
- Not UPF
- Don't know/not sure

Healthiness

1	2	3	4	5	6	7	8	9	10
○	○	○	○	○	○	○	○	○	○

24

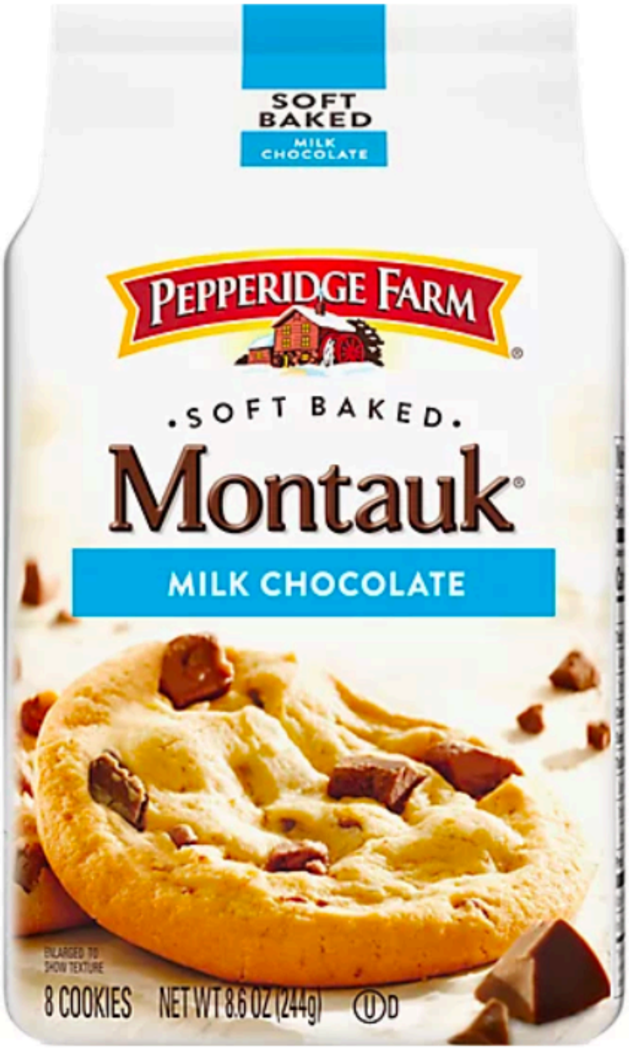

Nutrition Facts

8 Servings Per Container
Serving Size 1 Cookie (31g)

Amount per serving	
**Calories**	**140**
	**% Daily Value***
**Total Fat** 6g	**8%**
Saturated Fat 3.5g	**18%**
Trans Fat 0g	
**Cholesterol** 10mg	**3%**
**Sodium** 75mg	**3%**
**Total Carbohydrate** 21g	**8%**
Dietary Fiber 0g	**0%**
Total Sugars 11g	
Includes 11g Added Sugars	**22%**
**Protein** 1g	
Vitamin D 0mcg 0% • Calcium 10mg	0%
Iron 0.7mg 4% • Potassium 40mg	0%

*The % Daily Value (DV) tells you how much a nutrient in a serving of food contributes to a daily diet. 2,000 calories a day is used for general nutrition advice.

MADE FROM: ENRICHED WHEAT FLOUR (FLOUR, NIACIN, REDUCED IRON, THIAMINE MONONITRATE, RIBOFLAVIN, FOLIC ACID), MILK CHOCOLATE (SUGAR, COCOA BUTTER, SKIM MILK, CHOCOLATE, MILK FAT, DEXTROSE, SOY LECITHIN, VANILLA EXTRACT), FRUCTOSE, VEGETABLE OILS (PALM AND/OR SOYBEAN AND HYDROGENATED SOYBEAN), BROWN SUGAR (SUGAR, INVERT SUGAR, MOLASSES), BUTTER, CORN SYRUP SOLIDS, SUGAR, CONTAINS 2% OR LESS OF: EGGS, CORNSTARCH, BAKING SODA, SALT, NATURAL FLAVORS, CREAM OF TARTAR, AMMONIUM BICARBONATE.
CONTAINS: WHEAT, MILK, SOY, EGGS.

Processing Level

- ○ UPF
- ○ Not UPF
- ○ Don't know/not sure

Healthiness

1 2 3 4 5 6 7 8 9 10

Part 3

How confident were you in your ability to correctly identify ultra-processed foods?

1 (not at all confident)	2	3	4	5	6 (extremely confident)

What factors or information did you use to classify foods as ultra-processed or not ultra-processed?

What factors or information did you use to rate foods' healthiness?

Demographic Information

What is your age?

Please list the gender with which you currently identify.

- Male
- Female
- Non-binary
- Prefer not to say

What is your race/ethnicity? Check all that apply.

- White
- African-American
- Hispanic
- Asian or Pacific Islander
- Native American

☐ [ ] Other (please specify)

☐ Prefer not to answer

In which field do you currently work or study?

[▼]

What is your highest level of education completed?

○ High school
○ Some college (attended but left college)
○ Enrolled in college (currently a student)
○ College/university degree
○ Masters degree
○ Terminal degree (e.g. PhD, MD, PharmD, JD)

In what region of the U.S. do you currently reside?

○ Northeast (New England and Mid-Atlantic)
○ South/southeast
○ Midwest
○ West coast/southwest

Email

Thank you for taking time to complete our survey. If you would like to be entered into a drawing for one of four $50 Amazon gift cards, please copy the link below to your browser to enter your email address. This is completely optional - if you do not wish to enter the drawing, click forward to exit the survey.

https://forms.gle/N7bvyJHyCbS34vqw5

Powered by Qualtrics
